# LINC00312 increases gemcitabine sensitivity in nasopharyngeal carcinoma by recruiting PABPC1 and modulating PAX5-mediated transcription of LPLUNC1

**DOI:** 10.1038/s41698-026-01542-7

**Published:** 2026-06-17

**Authors:** Zhen Guo, ZhiMin Zhang, Huai Liu, Feng Liu, Pan Chen, BinSheng He, Hui Wang, JiaoYang Lu

**Affiliations:** 1 Hunan Provincial Key Laboratory of the Fundamental and Clinical Research on Functional Nucleic Acid, the First Clinical College and the First Affiliated Hospital of Changsha Medical University, Changsha Medical University, Changsha, China; 2 Hunan Provincial Key Laboratory of the Research and Development of Novel Pharmaceutical Preparations, Changsha Medical University, Changsha, China; 3 Hunan Provincial Key Laboratory of the Fundamental and Clinical Research on Multimodal Oncology, the First Clinical College and the First Affiliated Hospital of Changsha Medical University, Changsha Medical University, Changsha, China; 4 Department of Pharmacy, Guizhou University of Traditional Chinese Medicine, Guiyang, Guizhou China; 5 Hunan Key Laboratory of Translational Radiation Oncology, Department of Radiation Oncology, Hunan Cancer Hospital and The Affiliated Cancer Hospital of Xiangya School of Medicine, Central South University, Changsha, China; 6 Hunan Provincial Key Laboratory of the Regional Characteristic Traditional Chinese Medicine Resources and Ecological Agriculture, Changsha Medical University, Changsha, China; 7 Hunan Provincial Key Laboratory of the Traditional Chinese Medicine Agricultural Biogenomics, Changsha Medical University, Changsha, China

**Keywords:** Cancer, Cell biology, Molecular biology, Oncology

## Abstract

Gemcitabine resistance is an important factor in the treatment failure of nasopharyngeal carcinoma (NPC). LINC00312 has been reported to associate with chemotherapy toxicity and chemoradiotherapy response in NPC. However, the molecular mechanism by which LINC00312 regulates NPC sensitivity to gemcitabine is unclear. We found overexpression of LINC00312 or LPLUNC1 inhibited NPC cell proliferation, migration, invasion, and increased gemcitabine sensitivity. Mechanistically, LINC00312 bound to PABPC1 to increase PAX5 mRNA stability. PAX5 transcriptionally activated LPLUNC1 to inhibit the malignant phenotype of NPC and increase gemcitabine sensitivity. PAX5 depletion diminished the inhibitory effects of gemcitabine on cell viability, while LPLUNC1 overexpression restored chemosensitivity. Furthermore, PAX5 interacted with LINC00312 promoter to transcriptionally activate LINC00312. In turn, the LINC00312-PAX5 feedback axis upregulated LPLUNC1 to inhibit the progression of NPC and increase gemcitabine sensitivity in NPC. In conclusion, LINC00312 increased PAX5 mRNA stability by recruiting PABPC1, which promoted LPLUNC1 transcription to regulate sensitivity of NPC to gemcitabine. Meanwhile, PAX5 in turn transcriptionally activated LINC00312 to form a feedback axis, which further increased gemcitabine sensitivity.

## Introduction

Nasopharyngeal carcinoma (NPC) is one of the common malignant tumors in the head and neck. According to the global cancer statistics for 2022, there were about 120,416 new cases and 73,476 deaths of NPC^1^. The prevalence of NPC is very high in Southeast/East Asia, and China has the highest incidence rate^2^. At present, radiotherapy alone or in combination with chemotherapy is still the main treatment method for NPC^3^. However, due to inherent or acquired radiation/chemotherapy resistance in tumors, about 15-30% of advanced NPC patients will experience recurrence or metastasis as the disease progresses, which is the main cause of death for NPC. Recent efforts to overcome such resistance have highlighted the importance of targeting specific molecular pathways. For example, afatinib was shown to reverse epithelial-mesenchymal transition (EMT) and enhance radiosensitivity in NPC by inhibiting the CD44-Stat3 axis, underscoring the potential of targeted interventions to modulate treatment response^4^.

As a new type of antineoplastic drug, gemcitabine has good efficacy on NPC in terms of single-agent chemotherapy, combined chemotherapy, or combined radiotherapy, and the toxicity and side effects are mild^5,6^. At present, cisplatin combined with gemcitabine is the first-line treatment for recurrent/metastatic NPC, with a median progression-free survival of about 7 months and a five-year overall survival rate of less than 20%^7,8^. These outcomes underscore the persistent challenge of chemoresistance and highlight the urgent need to identify novel molecular targets that can sensitize tumors to existing therapies. In this context, the discovery and validation of molecular signatures associated with treatment resistance have gained increasing attention, as exemplified by studies in other cancers that leverage multi-omics approaches to identify prognostic biomarkers and therapeutic targets. In conclusion, cisplatin/gemcitabine resistance poses a serious threat to the quality of life and prognosis of NPC patients. Therefore, elucidating the molecular mechanisms of gemcitabine resistance in NPC is of great significance for the individualized treatment of NPC patients.

Long noncoding RNAs (lncRNAs) play critical roles in regulating cellular pathophysiological processes and are closely associated with the onset and progression of cancer^9–13^. Recently, several lncRNAs have been reported to participate in the progression of NPC by regulating DNA damage repair, mitochondrial function, chromatin remodeling, and competitive endogenous RNAs^14–17^. LINC00312 plays a key role as a tumor suppressor gene in many cancers, including NPC. The genetic variation of LINC00312 is closely related to the susceptibility and the risk of chemotherapy toxic side effects in NPC patients^18,19^. NPC patients with high expression level of LINC00312 had better prognosis, and LINC00312 suppressed radiotherapy resistance by inhibiting DNA repair in NPC^20^. Furthermore, Zhang et al found that overexpression of LINC00312 increased the sensitivity of ovarian cancer to cisplatin, while knockdown of LINC00312 exerted the opposite effect^21^. However, there were no studies on LINC00312 regulating gemcitabine resistance in NPC.

Long palate, lung, and nasal epithelial cell clone 1 (LPLUNC1), also known as BPIFB1, is an immune molecule with anti-inflammatory and bactericidal properties present in nasopharyngeal respiratory epithelial cells^22^. Numerous studies demonstrated that LPLUNC1 performed an influential function in the pathogenesis of NPC. For example, overexpression of LPLUNC1 elevated p53 and reduced c-Myc expression in NPC cells, which reduced glycolysis in NPC^23^. LPLUNC1 attenuated NPC progression by inhibiting PHB1 ubiquitination to attenuate NF-κB activity^24^. LPLUNC1 inhibited the migration and invasion of NPC by interacting with VTN and VIM^25^. Additionally, LPLUNC1 blocked the immune escape of NPC via inhibiting PD-L1 expression^26^. Meanwhile, LPLUNC1 also suppressed VTN-mediated radiation resistance in NPC^27^. At present, the relationship between LPLUNC1 and chemotherapy resistance has not been reported.

The aim of this research is to examine the effect of LINC00312 and LPLUNC1 on gemcitabine resistance in NPC, and explore the molecular mechanism by which LINC00312 regulates LPLUNC1, enriching the regulatory mechanism of LINC00312 in chemosensitivity of NPC.

## Results

### LINC00312 and LPLUNC1 were downregulated in NPC and have a positive correlation

**Fig. 1:**
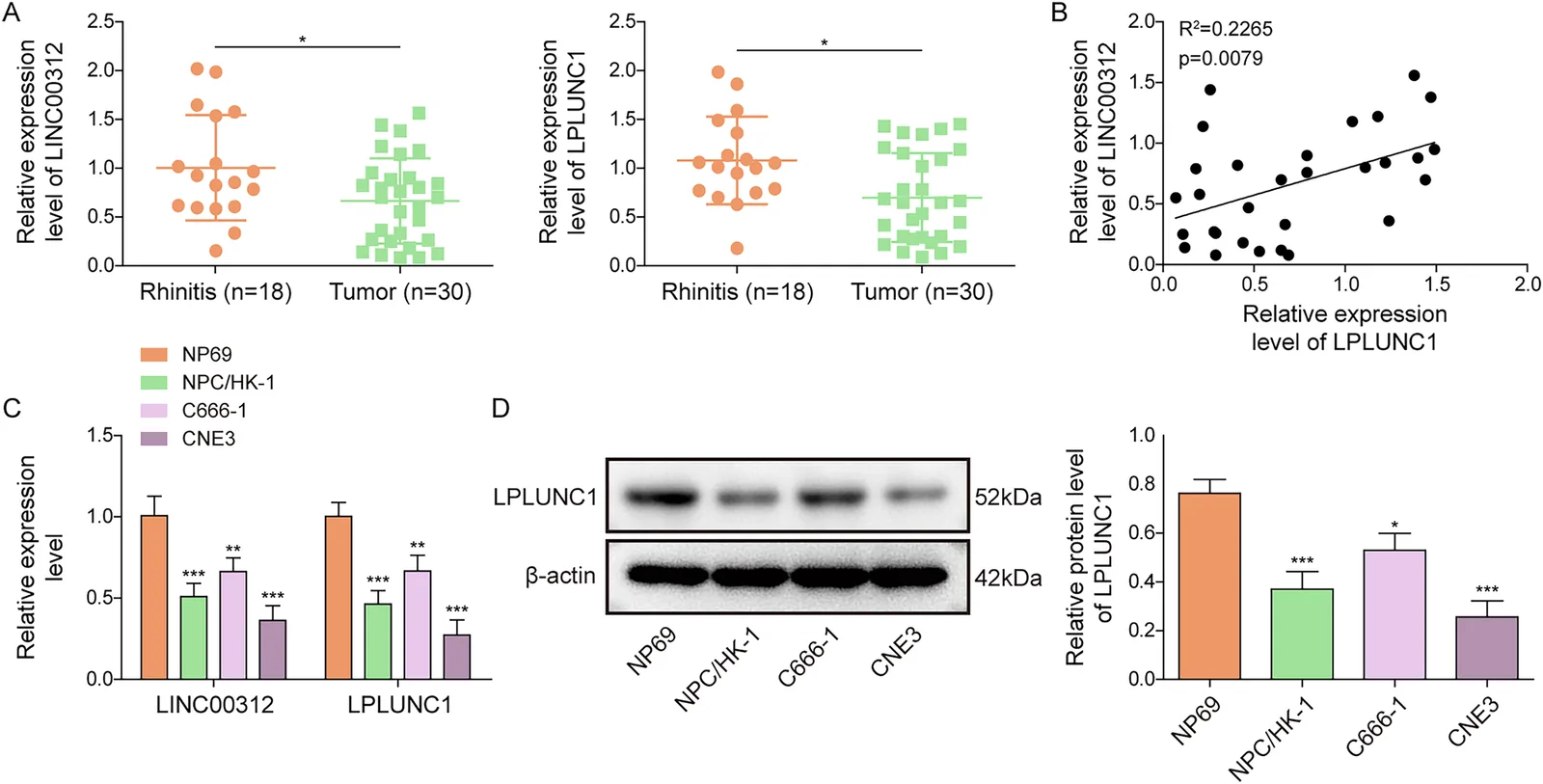
LINC00312 and LPLUNC1 were downregulated in NPC and have a positive correlation. **A** RT-qPCR was applied to assess the expression of LINC00312 and LPLUNC1 in NPC tissues and rhinitis tissues. **B** Pearson’s correlation coefficient was utilized to analyze the correlation between LINC00312 and LPLUNC1. **C**, **D** RT-qPCR and western blot were used to detect the expressions of LINC00312 and LPLUNC1 in NP69, CNE3, NPC/HK-1, and C666-1 cells. *p < 0.05, **p < 0.01, ***p < 0.001.

To elucidate the functional roles of LINC00312 and LPLUNC1 in NPC, we analyzed clinical NPC tissues and rhinitis tissues. Quantitative analysis revealed that both LINC00312 and LPLUNC1 expression levels were significantly downregulated in NPC tissues compared to rhinitis tissues (Fig. 1A). Due to the deep anatomical location and occult early symptoms resulting in a limited window period for sample acquisition at diagnosis, coupled with the scarcity of patient samples meeting research criteria in clinical practice, the number of NPC clinical samples obtained in this study was limited. Building upon this, we performed additional data analysis utilizing the GEO database, which revealed that LPLUNC1 was downregulated in NPC tissues (Supplementary Fig. 1A). The expression of LINC00312 and LPLUNC1 was positively correlated in NPC (Fig. 1B & Supplementary Fig. 1B). Additionally, compared with normal nasopharyngeal epithelial cell NP69, the levels of LINC00312 and LPLUNC1 in NPC cells (CNE3, NPC/HK-1, and C666-1) were significantly decreased (Fig. 1C, D). Our findings further indicate that NPC patients exhibiting lower expression of LINC00312 and LPLUNC1 demonstrated significantly reduced survival compared to those with elevated expression of these genes (Supplementary Fig. 2A). These results suggest that both LINC00312 and LPLUNC1 may play tumor-suppressive roles in NPC pathogenesis.

### Overexpression of LINC00312 inhibited the proliferation, invasion, and migration of NPC cells and increased the sensitivity to gemcitabine

**Fig. 2:**
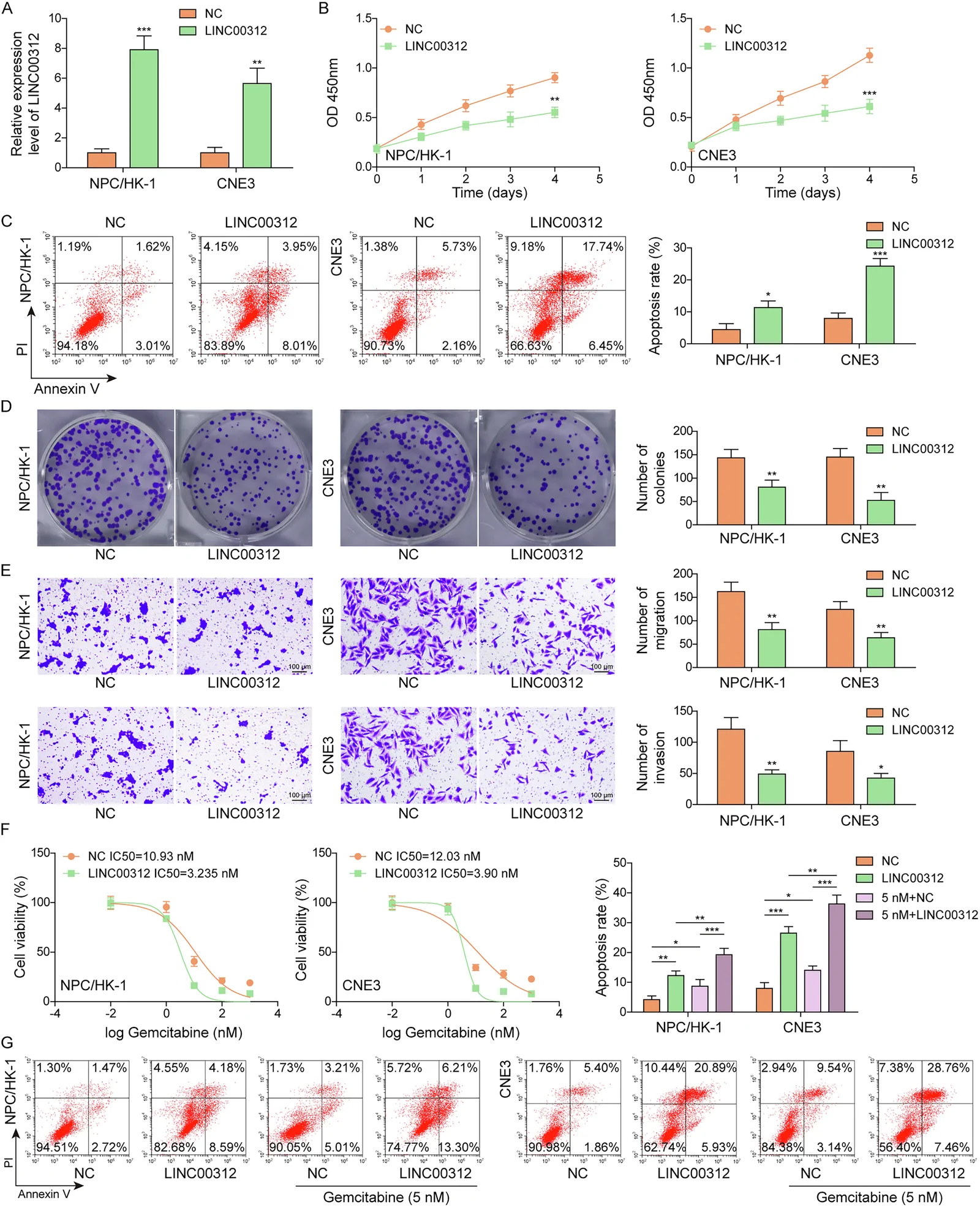
Overexpression of LINC00312 inhibited the proliferation, invasion and migration of NPC and increased the sensitivity to gemcitabine. NPC/HK-1 and CNE3 cells were transfected with NC (negative control for overexpressing vector) or LINC00312 overexpressing vector. **A** The expression of LINC00312 was examined by RT-qPCR. **B** Cell viability was tested by CCK-8. **C** Flow cytometry was employed to assess cell apoptosis. **D** The proliferative capacity of cells was measured by the colony formation assay. **E** Transwell assay was implemented to detect the invasion and migration abilities of cells (scale bar = 100 μm). **F** CCK-8 was adopted to calculate cellular viability after gemcitabine treatment. **G** After treatment with gemcitabine, cell apoptosis was assessed using flow cytometry. *p < 0.05, **p < 0.01, ***p < 0.001.

To explore the role of LINC00312 in modulating the malignant characteristics of NPC and its influence on gemcitabine sensitivity, we initially assessed LINC00312 expression levels in NPC tissues and cells subjected to gemcitabine treatment. The findings indicated that LINC00312 expression was significantly upregulated in gemcitabine-treated NPC samples and cells compared to untreated controls (Supplementary Fig. 2B, C). Furthermore, LINC00312 expression was notably reduced in gemcitabine-resistant NPC tissues relative to gemcitabine-sensitive counterparts (Supplementary Fig. 2D). Following these observations, LINC00312 overexpression vectors were transfected into CNE3 and NPC/HK-1 cell lines to induce LINC00312 overexpression in NPC models (Fig. 2A). Next, we observed that overexpression of LINC00312 decreased cell viability (Fig. 2B), weakened cell proliferation, invasion, and migration abilities, but increased the rate of apoptosis (Fig. 2C–E). To examine the effect of LINC00312 on chemosensitivity, cells were treated with increasing concentrations of gemcitabine. Dose-response analysis revealed that 5 nM gemcitabine induced approximately 50% reduction in viability (Fig. 2F), and this concentration was selected for subsequent apoptosis assays. LINC00312 overexpression also potentiated gemcitabine-induced apoptosis (Fig. 2G). It is important to highlight that our findings demonstrate LINC00312’s ability to increase the sensitivity of NPC cells to both cisplatin (DDP) and 5-fluorouracil (5-FU) (Supplementary Fig. 2E, F). This suggests that the modulatory effect of LINC00312 on chemotherapy responsiveness in NPC extends beyond gemcitabine. Collectively, these findings demonstrated that LINC00312 acts as a tumor suppressor in NPC by inhibiting malignant behaviors and enhancing chemotherapeutic sensitivity to gemcitabine.

### Overexpression of LPLUNC1 suppressed the proliferation, invasion, and migration of NPC and increased the sensitivity to gemcitabine

**Fig. 3:**
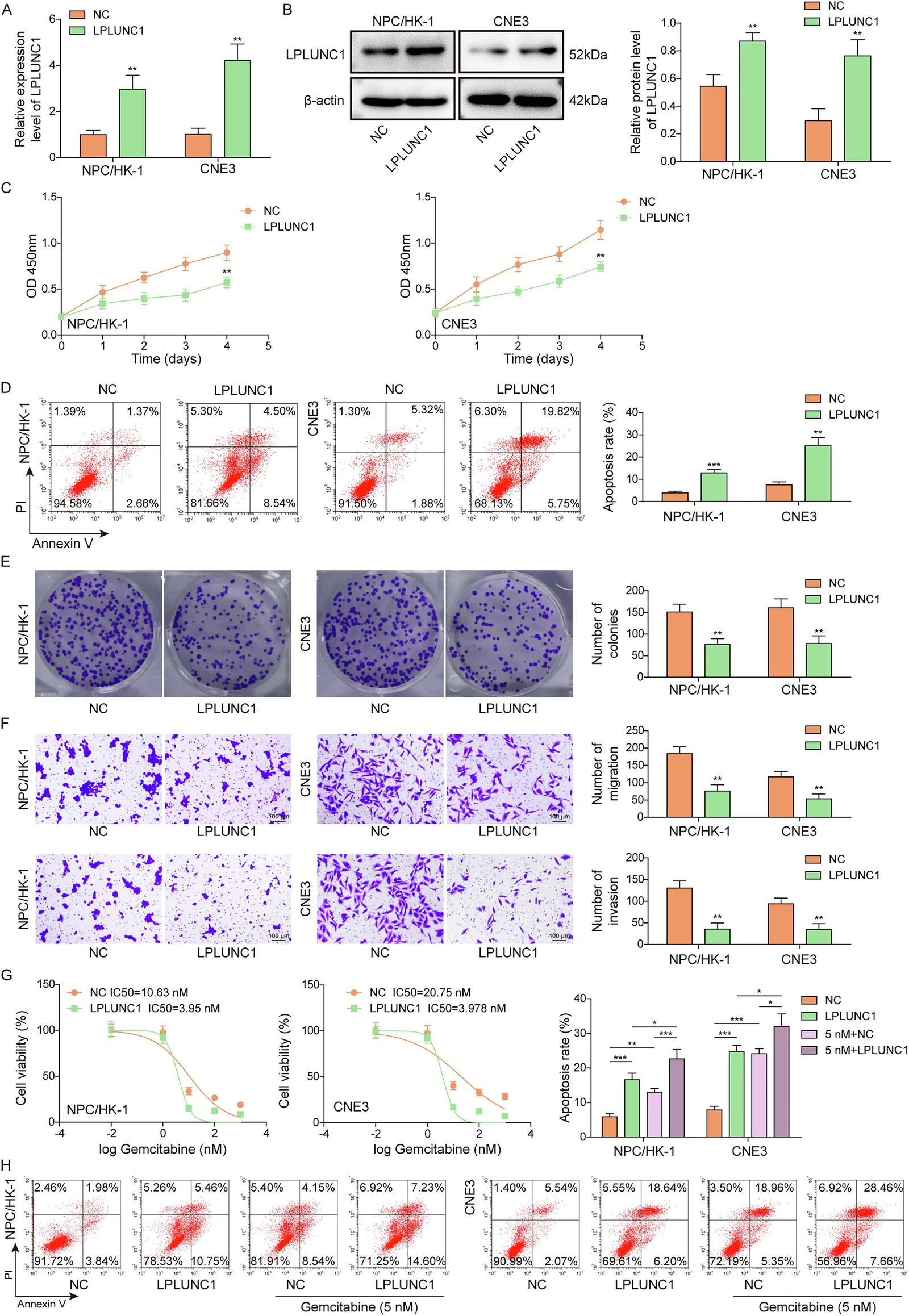
Overexpression of LPLUNC1 suppressed the proliferation, invasion, and migration of NPC and increased the sensitivity to gemcitabine. NPC/HK-1 and CNE3 cells were transfected with NC (negative control for overexpressing vector) or LPLUNC1 overexpressing vector. **A**, **B** The level of LPLUNC1 was evaluated using RT-qPCR and western blot. **C** CCK-8 was adopted to test the viability of cells. **D** Flow cytometry was utilized to analyze apoptosis. **E** Colony formation assay was deployed to test the proliferative capacity. **F** The number of migrating and invading cells was detected by transwell assay (scale bar = 100 μm). **G** After treatment with gemcitabine, the cell viability was examined by CCK-8. **H** After treatment with gemcitabine, cell apoptosis was estimated by flow cytometry. *p* < 0.05, **p < 0.01, ***p < 0.001.

Subsequently, we examined the function of LPLUNC1 in modulating the sensitivity of NPC cells to gemcitabine. Initially, clinical data revealed an upregulation of LPLUNC1 expression in NPC tissues following gemcitabine treatment (Supplementary Fig. 3A, B). Furthermore, LPLUNC1 levels were markedly reduced in gemcitabine-resistant NPC patient samples compared to those that were gemcitabine-sensitive (Supplementary Fig. 3C, D). This expression pattern aligns with the previously described profile of LINC00312. Thereafter, we overexpressed LPLUNC1 in the CNE3 and NPC/HK-1 cell lines (Fig. 3A, B). Functional assays revealed that elevated LPLUNC1 expression markedly suppressed cell viability, proliferation, migration, and invasion, while simultaneously promoting apoptosis (Fig. 3C–F). Furthermore, LPLUNC1 overexpression enhanced the sensitivity of NPC cells to gemcitabine and further promoted gemcitabine-induced apoptosis (Fig. 3G, H). Collectively, these findings demonstrated that LPLUNC1 attenuated the malignant behaviors of NPC cells and increased NPC cell sensitivity to gemcitabine.

### PAX5 transcriptionally activated LPLUNC1

**Fig. 4:**
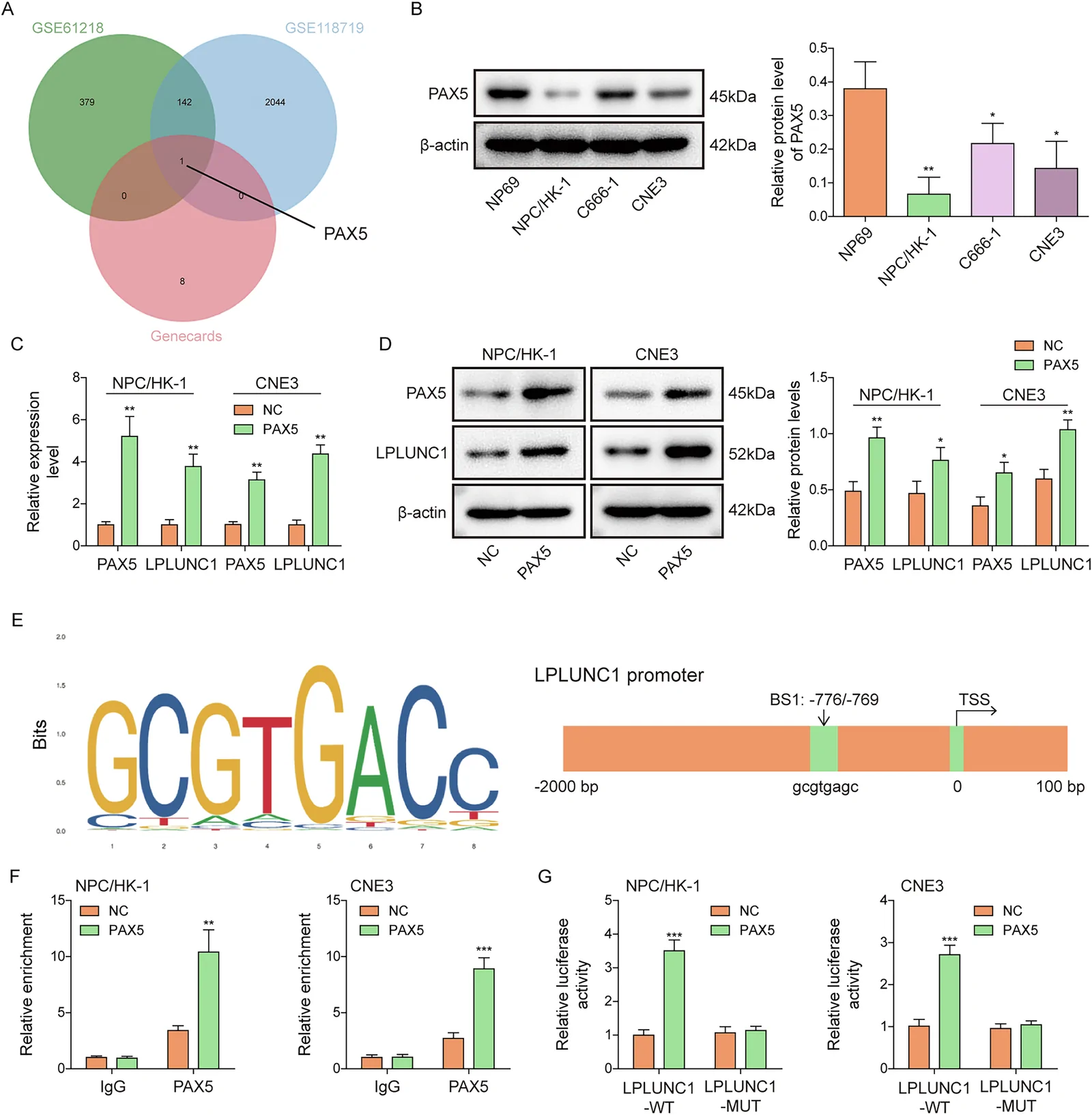
PAX5 transcriptionally activated LPLUNC1. **A** Venn diagram was adopted to show the intersection of genes downregulated in both the GSE118719 and GSE61218 databases and transcription factors on the LPLUNC1 promoter according to the Genecards database. **B** Western blot was used to examine the expression of PAX5 in NP69, CNE3, NPC/HK-1, and C666-1 cells. **C**, **D** NPC/HK-1 and CNE3 cells were transfected with NC (negative control for overexpressing vector) or PAX5 overexpressing vector. RT-qPCR and western blot were employed to test PAX5 and LPLUNC1 levels. **E** The interaction between PAX5 and LPLUNC1 promoter was predicted by JASPAR. **F** The binding of PAX5 and LPLUNC1 promoter in CNE3 and NPC/HK-1 cells was verified by ChIP. **G** Dual-luciferase reporter assay was employed to measure the interaction of PAX5 and LPLUNC1 promoter in NPC cells. *p < 0.05, **p < 0.01, ***p < 0.001.

To elucidate the upstream regulatory mechanism of LPLUNC1 in NPC, we analyzed downregulated genes from the GSE61218 and GSE118719 datasets and intersected them with transcription factors predicted to bind the LPLUNC1 promoter (GeneCards database), and paired box 5 (PAX5) was screened out (Fig. 4A). Next, we investigated PAX5 expression in NPC specimens and observed marked downregulation of PAX5 in clinical and cell samples (Supplementary Fig. 3E & Fig. 4B). Analysis of the GEO database also revealed a reduced expression of PAX5 in NPC tissues (Supplementary Fig. 1C). We then assessed PAX5 expression levels in NPC tissues subjected to gemcitabine treatment. The findings indicated that PAX5 expression was significantly upregulated in gemcitabine-treated NPC samples and cells compared to untreated controls (Supplementary Fig. 3A-B, F). Furthermore, PAX5 expression exhibited a positive correlation with the levels of LINC00312 and LPLUNC1 in NPC tissues (Supplementary Fig. 1D & Supplementary Fig. 3G). Subsequent prognostic analyses demonstrated that patients exhibiting reduced PAX5 expression experienced significantly shorter survival durations (Supplementary Fig. 2A). Collectively, these findings suggest a robust association between diminished PAX5 expression and the advancement of NPC.

Overexpression of PAX5 led to a significant upregulation of both PAX5 and LPLUNC1 at the mRNA and protein levels (Fig. 4C, D). Further bioinformatic analysis (JASPAR database) predicted PAX5 binding sites within the LPLUNC1 promoter (Fig. 4E). ChIP assay validated that PAX5 binds to LPLUNC1 promoter, and was strengthened by overexpression of PAX5 (Fig. 4F). Meanwhile, dual-luciferase reporter assays confirmed that PAX5 overexpression significantly increased luciferase activity in NPC cells transfected with wild-type LPLUNC1 promoter constructs (LPLUNC1-WT), but not mutant (LPLUNC1-MUT) constructs (Fig. 4G). Together, these findings demonstrate that PAX5 directly binds the LPLUNC1 promoter to activate its transcription.

### PAX5 suppressed NPC malignancy and increased gemcitabine sensitivity by modulating LPLUNC1

**Fig. 5:**
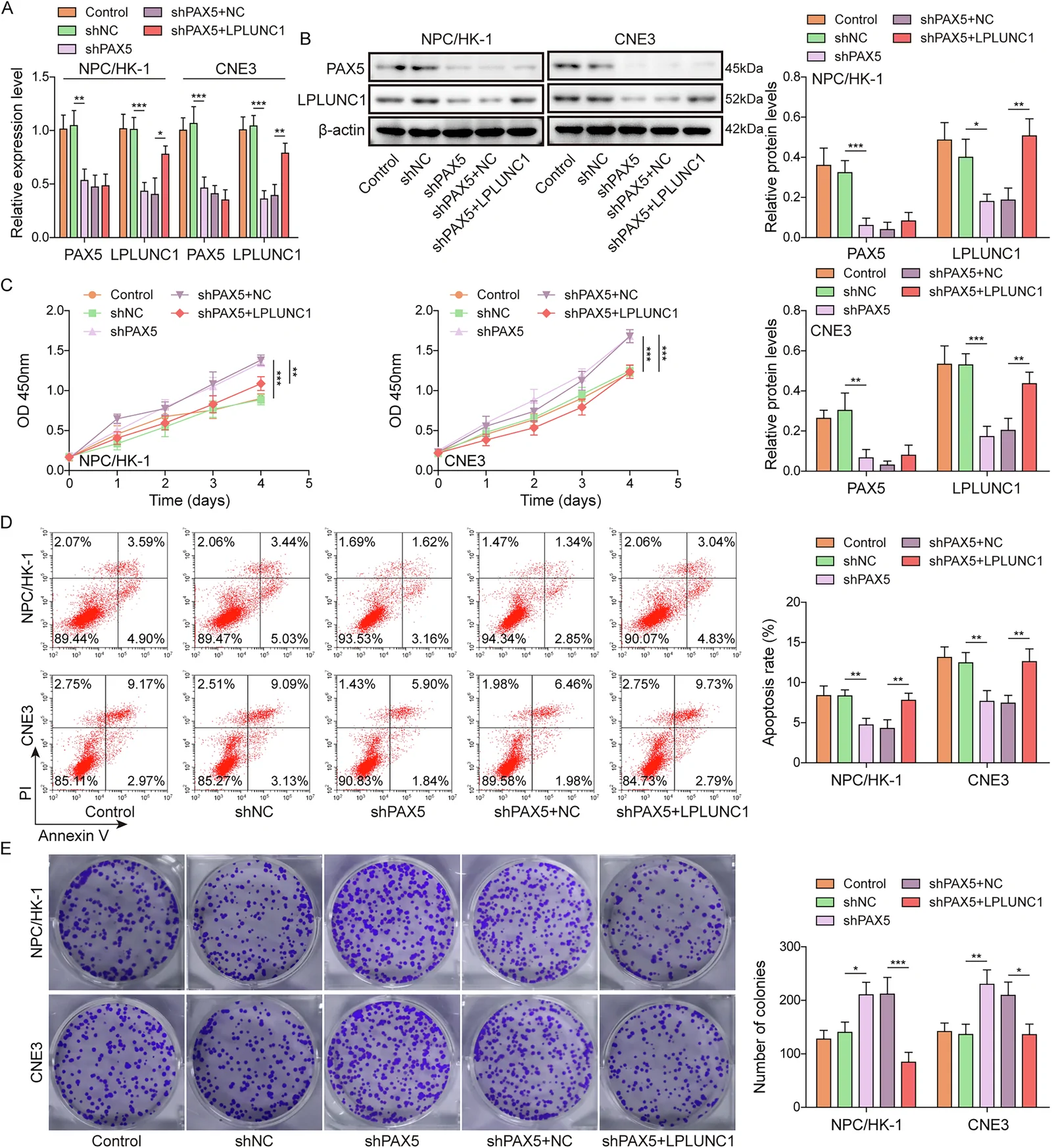

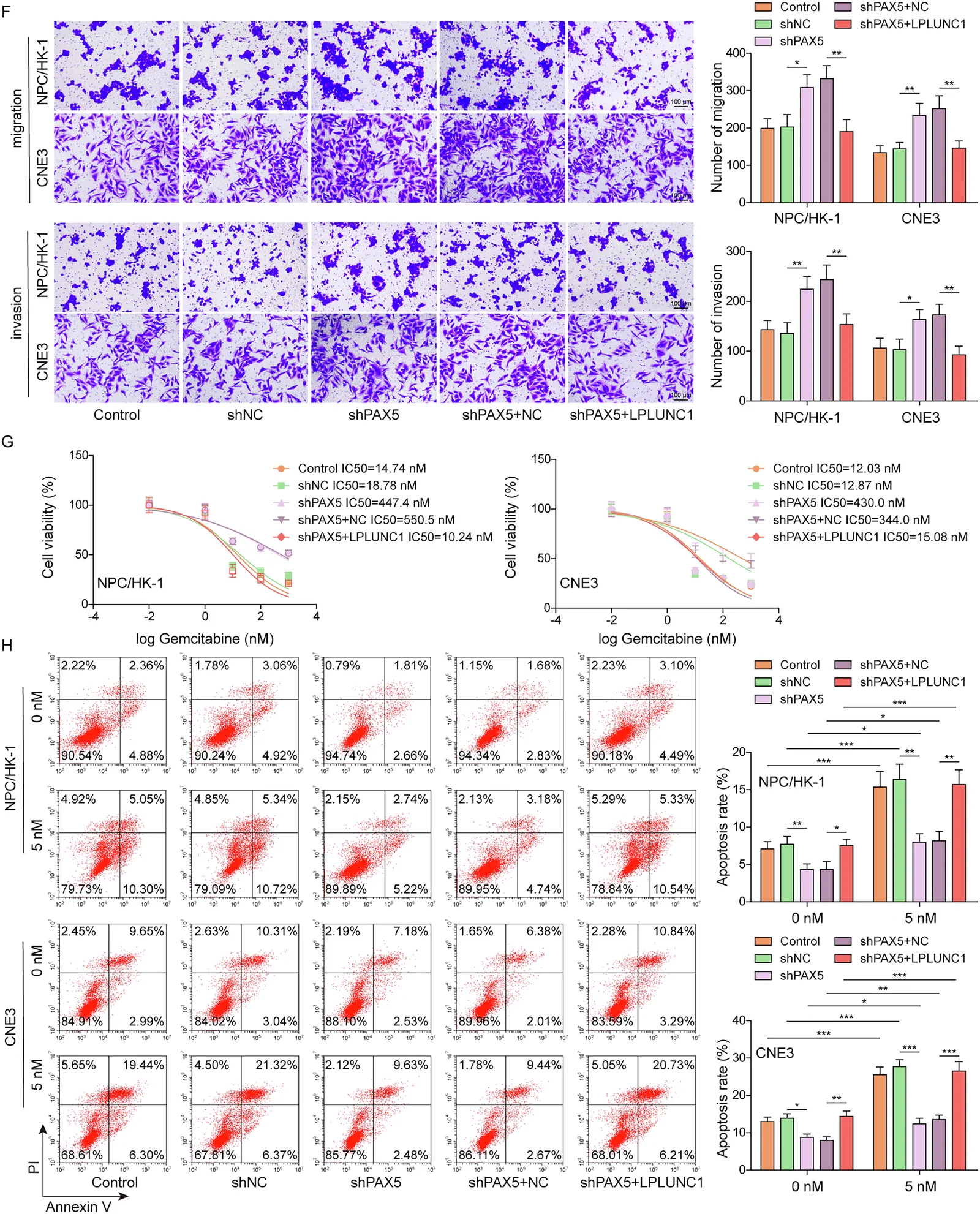
PAX5 suppressed NPC malignancy and increased gemcitabine sensitivity by modulating LPLUNC1. NPC/HK-1 and CNE3 cells were transfected with shPAX5 or/and LPLUNC1 overexpression vector. shNC means negative control for shRNA, while NC means negative control for the overexpressing vector. “Control” refers to cells that have not undergone any genetic manipulation. **A**, **B** RT-qPCR and western blot were adopted to measure the levels of PAX5 and LPLUNC1. **C** Cell viability was assayed using CCK-8. **D** Flow cytometry was used to evaluate apoptosis. **E** Cell proliferation capacity was estimated by clone formation assays. **F** Transwell assay was applied to test the migrative and invasive ability of cells (scale bar = 100 μm). **G**, **H** CCK-8 and flow cytometry were employed to evaluate the cells’ viability and apoptosis after treatment with gemcitabine. *p < 0.05, **p < 0.01, ***p < 0.001.

To elucidate the functional role of PAX5-LPLUNC1 regulation in the progression of NPC and its response to gemcitabine treatment, we initially assessed PAX5 expression levels in NPC tissue and cells associated with gemcitabine therapy. Our analysis revealed that PAX5 expression was upregulated in gemcitabine-treated NPC tissues and cells compared to untreated counterparts (Supplementary Fig. 2C & Supplementary Fig. 3A-B, F). Moreover, PAX5 expression was markedly reduced in gemcitabine-resistant tissues relative to gemcitabine-sensitive tissues (Supplementary Fig. 3C, D). In light of these observations, we generated CNE3 and NPC/HK-1 cells with PAX5 knockdown, LPLUNC1 overexpression, or both. And the data showed that knockdown of PAX5 markedly reduced both PAX5 and LPLUNC1 expression, while LPLUNC1 overexpression rescued this effect (Fig. 5A, B). Moreover, knockdown of PAX5 led to a promotion of cell proliferation, invasion, and migration, while a reduction in cell apoptosis, and overexpression of LPLUNC1 relieved the above phenomenon by knockdown of PAX5 (Fig. 5C–F). Moreover, PAX5 depletion diminished the sensitivity of NPC cells to gemcitabine and attenuated gemcitabine-induced apoptosis, whereas LPLUNC1 overexpression restored chemosensitivity (Fig. 5G, H). Notably, the overexpression of PAX5 in NPC cells did not alter their sensitivity to cisplatin and 5-FU, suggesting that PAX5 specifically modulates the response to gemcitabine treatment (Supplementary Fig. 3H–I). The findings presented above indicate that PAX5 constrained NPC progression and enhanced gemcitabine sensitivity by regulating LPLUNC1.

### LINC00312 increased PAX5 mRNA stability by interacting with PABPC1

**Fig. 6:**
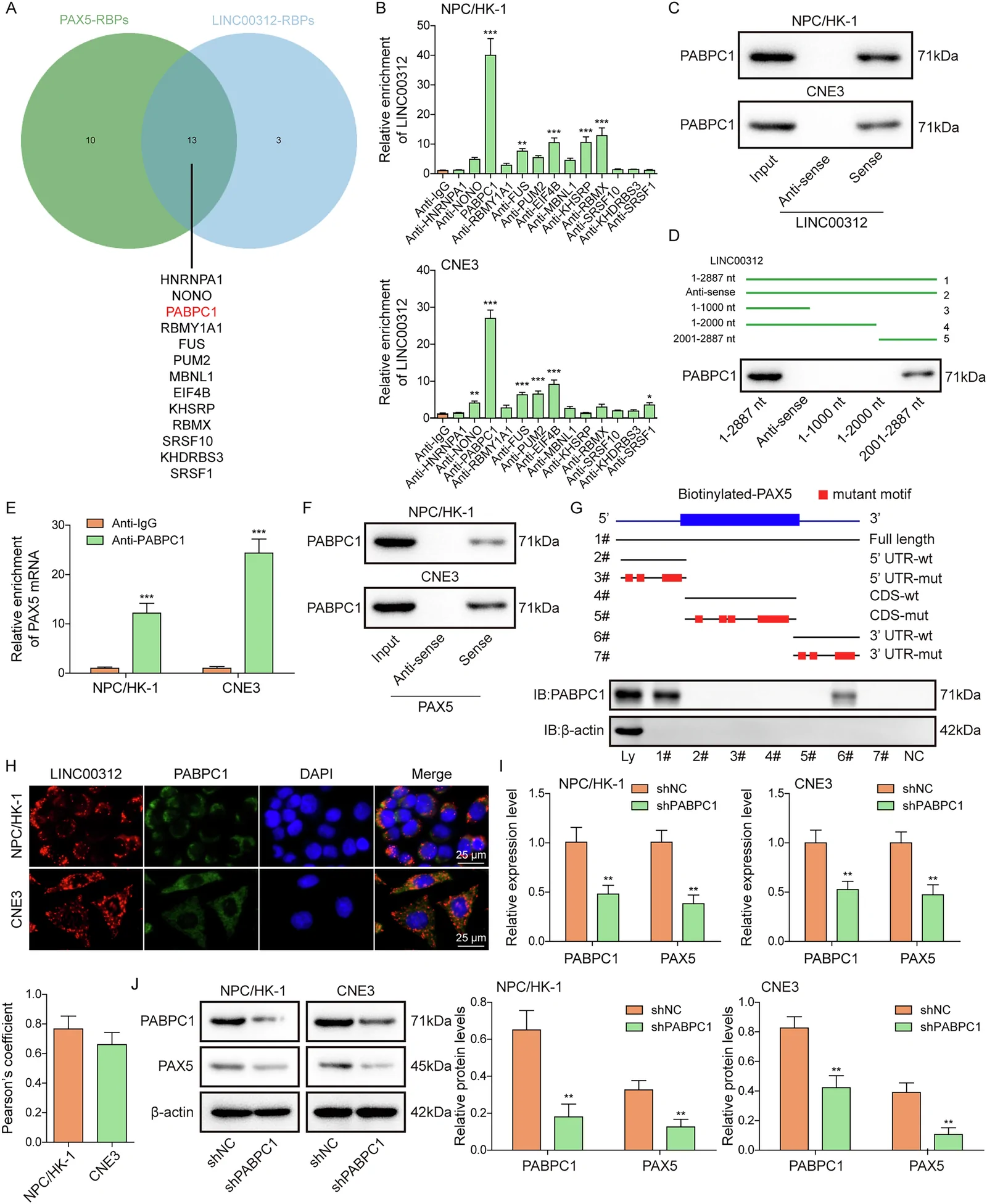

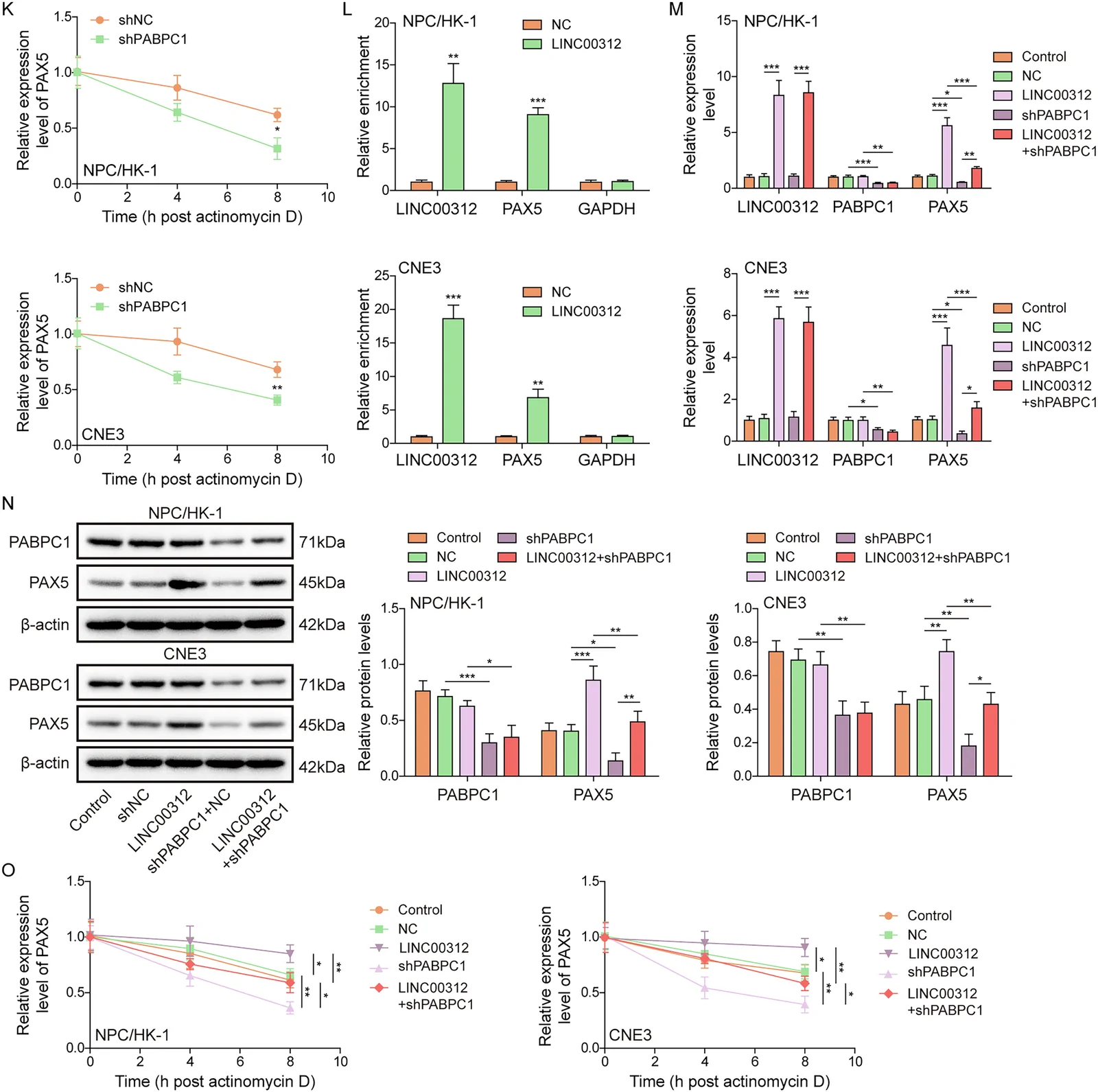
LINC00312 increased PAX5 mRNA stability by interacting with PABPC1. **A** The Rbpdb website was utilized to predict RBP proteins that bind to LINC00312 and PAX5 mRNA. **B** RIP assay was employed to test the binding of LINC00312 to RBPs in NPC/HK-1 and CNE3 cells. **C** RNA pulldown assay was adopted to verify the interaction of LINC00312 with the PABPC1 protein. **D** 293 T cells were used to construct various truncations of LINC00312, and the interaction between these LINC00312 truncations and PABPC1 was analyzed using RNA pulldown. **E**, **F** RNA pulldown and RIP were employed to validate the binding of PAX5 mRNA to PABPC1 protein in NPC/HK-1 and CNE3 cells. **G** Various truncations of PAX5 mRNA (PAX5-3′UTR, PAX5-CDS, PAX5-5′UTR) were transfected into 293 T cells, and RNA pulldown was used to examine the interactions between PABPC1 and PAX5 mRNA truncations. **H** Localization of LINC00312 and PABPC1 in NPC/HK-1 and CNE3 cells was detected by FISH and IF (scale bar = 25 μm). **I**–**J** RT-qPCR and western blot were utilized to detect the levels of PABPC1 and PAX5 in NPC/HK-1 and CNE3 cells after knocking down PABPC1. shNC means negative control for shRNA, while NC means negative control for the overexpressing vector. “Control” refers to cells that have not undergone any genetic manipulation. **K** The mRNA stability of PAX5 was detected by RT-qPCR in NPC/HK-1 and CNE3 cells with PABPC1 knockdown after treatment with actinomycin D. **L** RIP assay was applied to test the binding of PAX5 mRNA to PABPC1 protein in NPC/HK-1 and CNE3 cells after overexpression of LINC00312. **M**, **N** NPC/HK-1, and CNE3 cells were transfected with LINC00312 overexpression vector and/or shPABPC1 vector. The expression levels of LINC00312, PABPC1, and PAX5 were measured by RT-qPCR and western blot. **O** RT-qPCR was adopted to assess PAX5 mRNA stability in NPC/HK-1 and CNE3 cells transfected with LINC00312 overexpression vector and/or shPABPC1 vector after actinomycin D treatment. *p < 0.05, **p < 0.01, ***p < 0.001.

LncRNAs have been reported to interact with RNA-binding proteins (RBPs) to regulate the stability of downstream mRNAs. To investigate whether LINC00312 regulates PAX5 mRNA stability by binding to RBPs, the Rbpdb website was utilized to identify potential RBPs that interact with both LINC00312 and PAX5 mRNA. Bioinformatics analysis revealed 13 common binding proteins for LINC00312 and PAX5, including HNRNPA1, NONO, poly (A) binding protein cytoplasmic 1 (PABPC1), RBMY1A1, FUS, PUM2, MBNL1, EIF4B, KHSRP, RBMX, SFRS13A, KHDRBS3, and SFRS1 (Fig. 6A). Subsequent RIP assay demonstrated significant enrichment of LINC00312 with anti-PABPC1 antibody compared to other antibodies, suggesting a preferential interaction between LINC00312 and PABPC1 (Fig. 6B). This interaction was further validated through RNA pull-down assays (Fig. 6C). To map the specific binding region, we generated truncated variants of LINC00312 (1-2287 nt, 1-1000 nt, 1-2000 nt, and 2001-2887 nt) and identified that the 2001-2887 nt fragment mediated the interaction with PABPC1 (Fig. 6D). Furthermore, RNA pulldown and RIP assays were applied to confirm that PAX5 mRNA interacted with the PABPC1 protein (Fig. 6E, F). Through domain mapping using PAX5 mRNA mutants (3’UTR, CDS, and 5’UTR), we determined that PABPC1 specifically binds to the 3’UTR region of PAX5 mRNA (Fig. 6G). Moreover, LINC00312 and PABPC1 were colocalized in the cytoplasm (Fig. 6H).

What’s more, knockdown of PABPC1 reduced both PABPC1 and PAX5 expression levels (Fig. 6I, J). Besides, knocking down PABPC1 decreased PAX5 mRNA stability following actinomycin D treatment (Fig. 6K). Notably, LINC00312 overexpression enhanced PABPC1-mediated enrichment of PAX5 mRNA (Fig. 6L). Further experiments showed that upregulation of LINC00312 greatly increased both LINC00312 and PAX5 levels without affecting PABPC1 expression, but knockdown of PABPC1 attenuated the levels of PABPC1 and PAX5, which counteracted the effects of LINC00312 overexpression on PAX5 (Fig. 6M, N). Consistent with these findings, LINC00312 overexpression increased PAX5 mRNA stability, and this effect was abolished by PABPC1 knockdown (Fig. 6O). Collectively, these results demonstrate that LINC00312 stabilizes PAX5 mRNA through a specific interaction with PABPC1 protein.

### LINC00312-PAX5 feedback axis upregulated LPLUNC1 to suppress NPC progression and enhance gemcitabine sensitivity

**Fig. 7:**
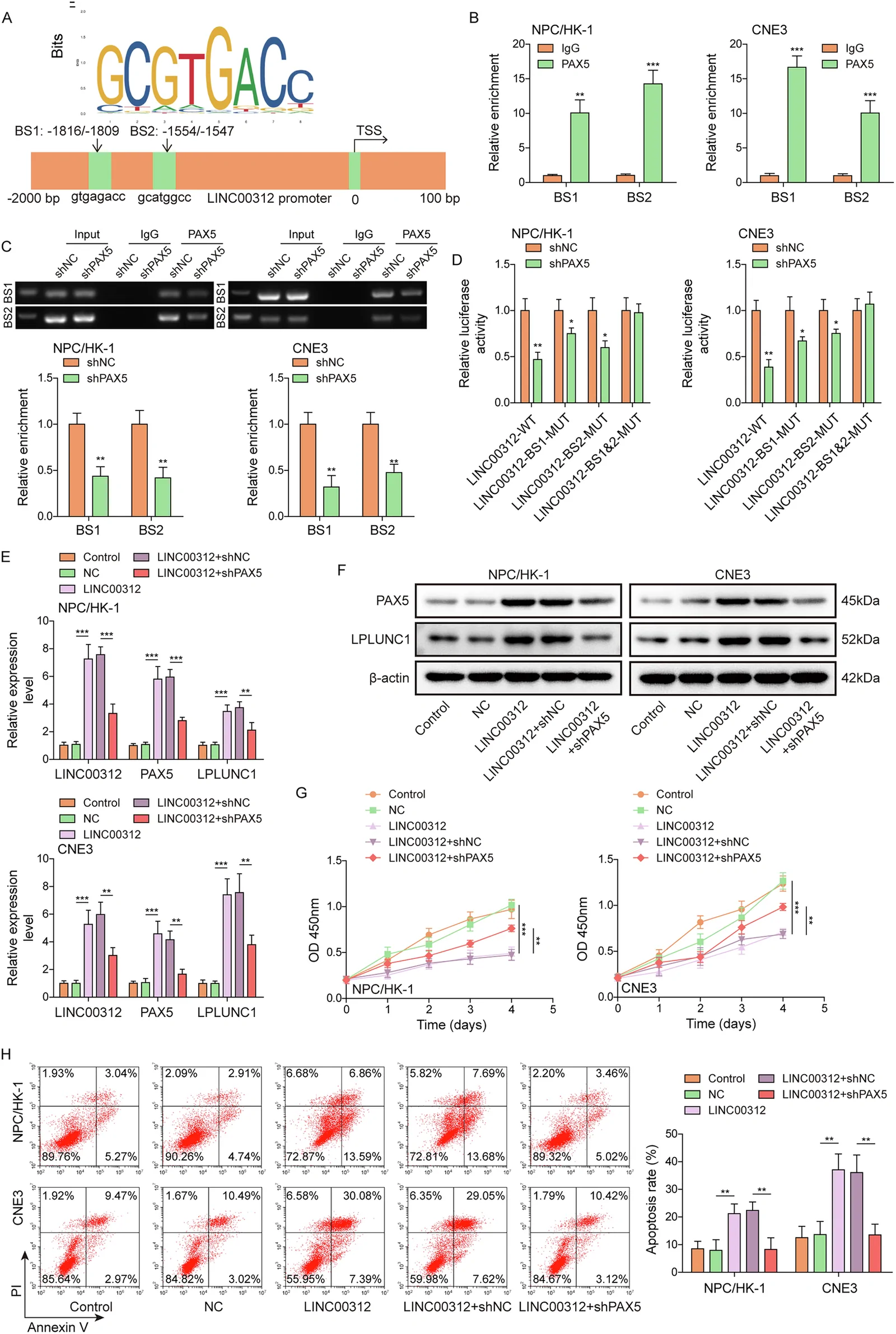

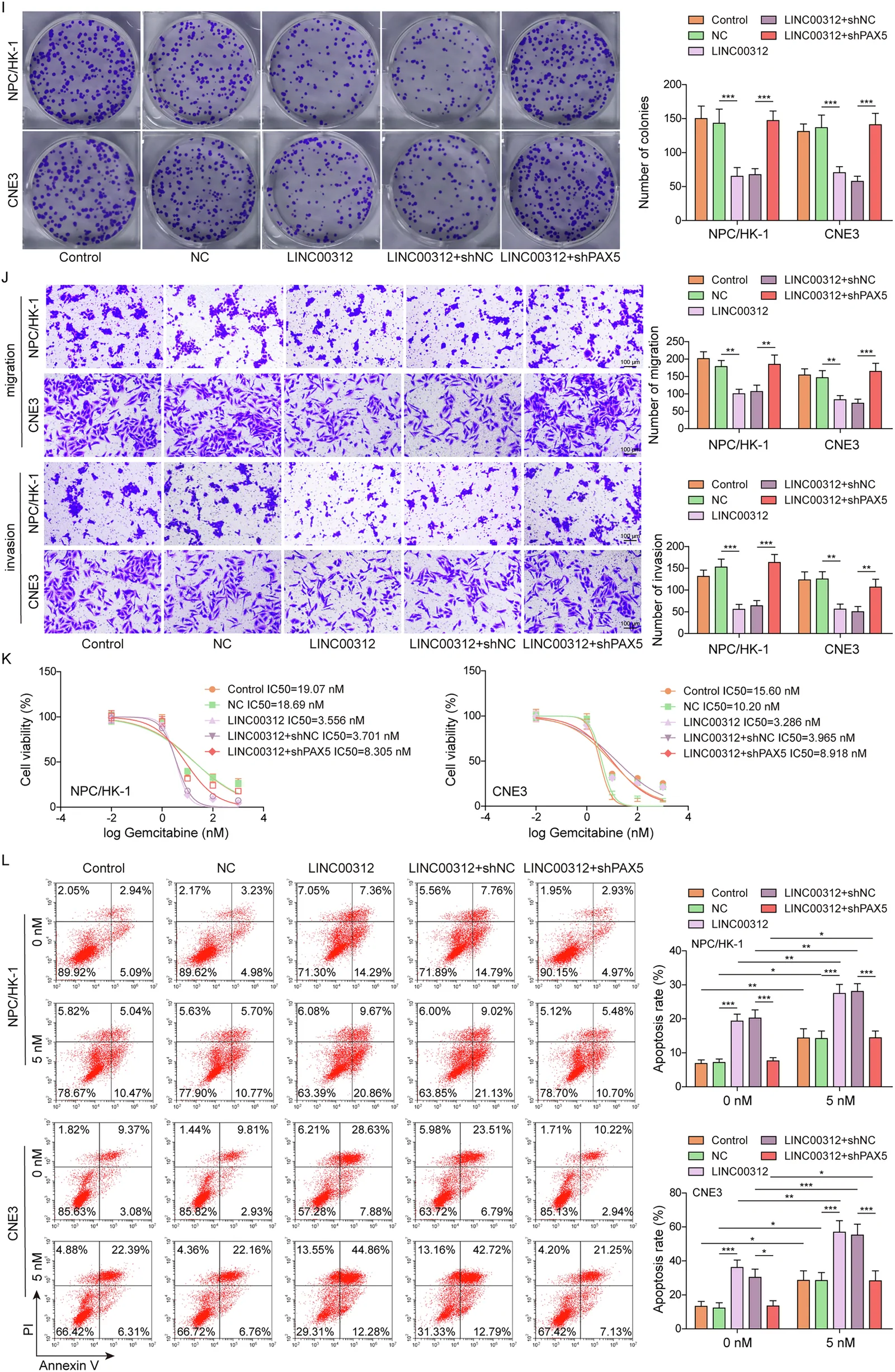
LINC00312-PAX5 feedback axis upregulated LPLUNC1 to suppress NPC progression and enhance gemcitabine sensitivity. **A** JASPAR was deployed to predict the interaction of PAX5 with LINC00312 promoter. **B** ChIP was applied to examine the binding of PAX5 to the LINC00312 promoter. **C**, **D** shNC (negative control for shRNA) or shPAX5 vector was transfected into NPC/HK-1 and CNE3 cells. ChIP and dual-luciferase reporter assays were used to detect PAX5 interaction with LINC00312 promoters. **E**, **F** NPC/HK-1 and CNE3 cells were transfected with LINC00312 and/or the shPAX5 vector. NC means negative control for the overexpressing vector. “Control” refers to cells that have not undergone any genetic manipulation. Cell viability and apoptosis were detected by CCK-8 and flow cytometry, respectively. **G**, **H** RT-qPCR and western blot were used to assess the expression levels of LINC00312, PAX5, and LPLUNC1. **I**–**J** Colony formation and transwell assay were used to assess cell proliferation, migration, and invasion (scale bar = 100 μm). **K**, **L** CCK-8 and flow cytometry were utilized to determine NPC/HK-1 and CNE3 cell viability and apoptosis after treatment with gemcitabine. *p < 0.05, **p < 0.01, ***p < 0.001.

Notably, the JASPAR database predicted that PAX5 bound to two specific sites (BS1 and BS2) on the LINC00312 promoter (Fig. 7A). This interaction was validated by ChIP experiment, where anti-PAX5 antibody exhibited significant enrichment at the LINC00312 promoter regions BS1 and BS2 compared to IgG control (Fig. 7B). Knockdown of PAX5 reduced the enrichment of anti-PAX5 antibody to LINC00312 promoter BS1 and BS2, as evidenced by decreased enrichment in ChIP assays (Fig. 7C). Consistent with this, dual-luciferase reporter assays demonstrated that both the BS1 and BS2 sites contribute functionally to PAX5-mediated transcriptional regulation of LINC00312, and the regulatory effect of PAX5 is completely abrogated only when both sites are inactivated (Fig. 7D).

To further investigate the functional consequences of this regulatory axis, we transfected CNE3 and NPC/HK-1 cells with PAX5 knockdown and/or LINC00312 overexpression vector. Intriguingly, overexpression of LINC00312 increased the expression of LINC00312, PAX5, and LPLUNC1, whereas PAX5 knockdown had the opposite effect and abolished the stimulatory impact of LINC00312 overexpression on LINC00312, PAX5, and LPLUNC1 (Fig. 7E, F). Functionally, PAX5 silencing promoted NPC cell proliferation, invasion, and migration while suppressing apoptosis. Conversely, LINC00312 overexpression counteracted the above phenomenon by knockdown of PAX5 (Fig. 7G–J). Additionally, knockdown of PAX5 attenuated the cytotoxic effects of gemcitabine, reducing its ability to suppress cell viability and induce apoptosis. However, LINC00312 overexpression aggravated the effect of gemcitabine, which was reversed by the knockdown of PAX5 (Fig. 7K, L). In summary, our findings establish a LINC00312-PAX5 positive feedback loop that upregulates LPLUNC1, thereby suppressing NPC malignant progression and increasing chemosensitivity to gemcitabine.

### LINC00312-PAX5 feedback axis upregulated LPLUNC1 to inhibit NPC tumor growth and increase gemcitabine sensitivity in vivo

**Fig. 8:**
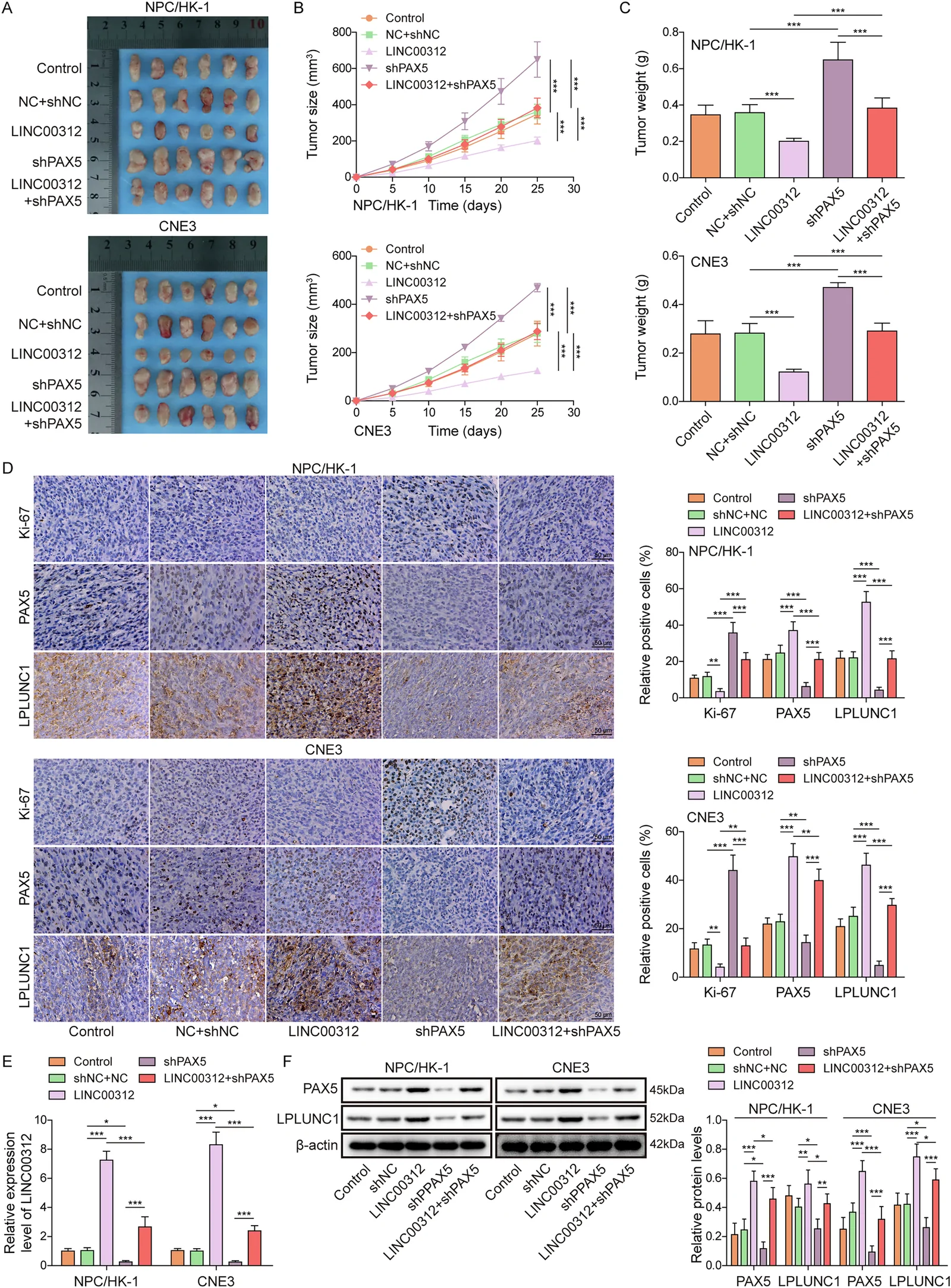
LINC00312-PAX5 feedback axis upregulated LPLUNC1 to inhibit NPC tumor growth and increase gemcitabine sensitivity in vivo*.* NPC/HK-1 and CNE3 cells with LINC00312 overexpression and/or PAX5 knockdown lentivirus were injected subcutaneously into nude mice, and mice were periodically injected intraperitoneally with gemcitabine. shNC means negative control for shRNA, while NC means negative control for the overexpressing vector. “Control” refers to cells that have not undergone any genetic manipulation. **A**–**C** Tumor size, weight, and volume changes were recorded. **D** IHC was applied to determine the levels of Ki67, PAX5, and LPLUNC1 (scale bar = 50 μm). **E** The expression level of LINC00312 was tested using RT-qPCR. **F** Western blot was used to estimate the levels of PAX5 and LPLUNC1. *p < 0.05, **p < 0.01, ***p < 0.001.

To further demonstrate whether the LINC00312-PAX5 feedback axis regulated NPC progression and gemcitabine sensitivity through LPLUNC1 in vivo, CNE3 cells or NPC/HK-1 cells stably overexpressing LINC00312 and/or knocking down PAX5 were subcutaneously injected into the right dorsal midline of mice. Consistent with the in vitro data, LINC00312 overexpression significantly suppressed tumor growth, as evidenced by reduced tumor volume and weight. Conversely, PAX5 knockdown promoted tumor progression and abrogated the tumor-suppressive effects of LINC00312 (Fig. 8A–C). Further immunohistochemical, qPCR, and western blot assays revealed that LINC00312 overexpression downregulated Ki67 while upregulating LINC00312, PAX5, and LPLUNC1 expression in tumor tissues (Fig. 8D–G). In contrast, knockdown of PAX5 showed the opposite trend and reversed the phenomenon caused by the overexpression of LINC00312 (Fig. 8D–G). Collectively, these in vivo results suggested that the LINC00312-PAX5 feedback axis upregulated LPLUNC1 to inhibit NPC tumor growth and increase gemcitabine sensitivity in vivo.

## Discussion

LncRNAs play pivotal regulatory roles in tumorigenesis and chemotherapy resistance, and targeting lncRNAs has emerged as a highly promising therapeutic approach for cancers^28^. For example, lncRNA-NEF suppressed oxaliplatin resistance and epithelial-mesenchymal transition in colorectal cancer^29^. LINC00312 enhanced the therapeutic efficacy of cisplatin in ovarian cancer by facilitating apoptosis^21^. A targeted nanocomplex carrying siRNA against MALAT1 could sensitize glioblastoma to temozolomide^30^. In this research, we found that LINC00312 is downregulated in NPC and functions as a prognostic indicator of unfavorable outcomes in NPC patients. Furthermore, overexpression of LINC00312 was shown to suppress the malignant characteristics of NPC cells and increase their sensitivity to gemcitabine treatment.

Many studies have pointed out that lncRNA acts as a molecular scaffold to promote the binding of RBP to mRNA, thereby regulating the stability of mRNA. As validated by RNA pull-down and RIP assay, we identified PABPC1 as a new binding protein of LINC00312. PABPC1 is a multifunctional RNA-binding protein that is mainly involved in the regulation of mRNA translation and stability^31^. PABPC1 enhanced BST2 mRNA stability in gastric cancer^32^. In esophageal squamous cell carcinoma, PABPC1 increased IFI27 mRNA stability by interacting with eIF4G^33^. Su et al found that PABPC1 increased BDNF-AS stability by interacting with BDNF-AS to inhibit the malignant progression of glioblastoma cells^34^.

Extensive studies have demonstrated that lncRNAs and circRNAs modulate downstream gene expression through interactions with PABPC1. For instance, circPTK2 promoted prostate cancer metastasis and gemcitabine resistance by regulating SETDB1 mRNA stability via PABPC1^35^. LncRNA TRPM2-AS enhanced gallbladder cancer angiogenesis by suppressing NUMB mRNA stability through PABPC1^36^. LncRNA WDR11-AS1 directly interacted with PABPC1 to stabilize SOX9 expression in osteoarthritis^37^. LncRNA PAXIP1-AS1 inhibited gastric cancer metastasis and progression by destabilizing PAK1 mRNA in a PABPC1-dependent manner^38^. LncRNA PRBC induced autophagy in breast cancer cells by regulating PABPC1 nuclear translocation and PABPC1-mediated mRNA stabilization^39^.

Importantly, our preliminary data from the Rbpdb database indicated that both LINC00312 and PAX5 bound to PABPC1. The binding between PABPC1 and PAX5 mRNA was confirmed by RIP assay. Notably, PABPC1 knockdown reduced PAX5 mRNA stability, leading to decreased levels of both PAX5 transcripts and protein. LINC00312 overexpression increased PAX5 mRNA stability, and this effect was attenuated by PABPC1 knockdown. Therefore, we revealed for the first time that LINC00312 interacted with PABPC1 to increase the stability of PAX5 mRNA in NPC.

PAX5 is a member of the paired-box transcription factor family and is crucial for transcriptional activation of target genes. Numerous studies suggested that PAX5 played an oncogene role in many cancers and was also critical for cancer chemoresistance. For example, PAX5 facilitated LINE transcription to enhance cellular senescence^40^. PAX5 increased the sensitivity of esophageal squamous cell carcinoma to fluorouracil by activating p53 transcription^41^. PAX5 enhanced the function of the proton complex by activating the transcription of ELP5, thereby sensitizing cholangiocarcinoma cells to gemcitabine treatment^42^. It has been reported that PAX5 expression was markedly downregulated in NPC tissues, which was significantly associated with advanced clinical staging, lower tumor mutation burden, enhanced immune activation, elevated EBV relative expression levels, and poorer patient survival outcomes^43^ In the study, it was uncovered that PAX5 restrained NPC proliferation, invasion, and migration and increased gemcitabine sensitivity through transcriptionally activating LPLUNC1. In addition, we validated the direct binding of PAX5 to the promoters of LPLUNC1 and LINC00312 in NPC cell lines, and clarified the functional necessity of two binding sites, BS1 and BS2, through site‑specific mutagenesis. Furthermore, clinical sample analysis showed that the expression of LINC00312, PAX5, and LPLUNC1 was upregulated after gemcitabine treatment, whereas it was significantly downregulated in gemcitabine‑resistant tissues, with low expression associated with poor patient prognosis. GEO public data further supported their downregulation and positive correlation in NPC. Mechanistically, we found drug specificity of this axis: PAX5 overexpression enhanced sensitivity only to gemcitabine but not to 5‑FU and cisplatin, while LINC00312 overexpression enhanced sensitivity to both cisplatin and 5‑FU. Together, these results reveal a feedback regulatory loop formed by LINC00312/PAX5/LPLUNC1, whose dysfunction is closely associated with gemcitabine resistance and poor prognosis in NPC, providing new theoretical and clinical insights for targeting this axis to improve chemotherapeutic efficacy.

**Fig. 9:**
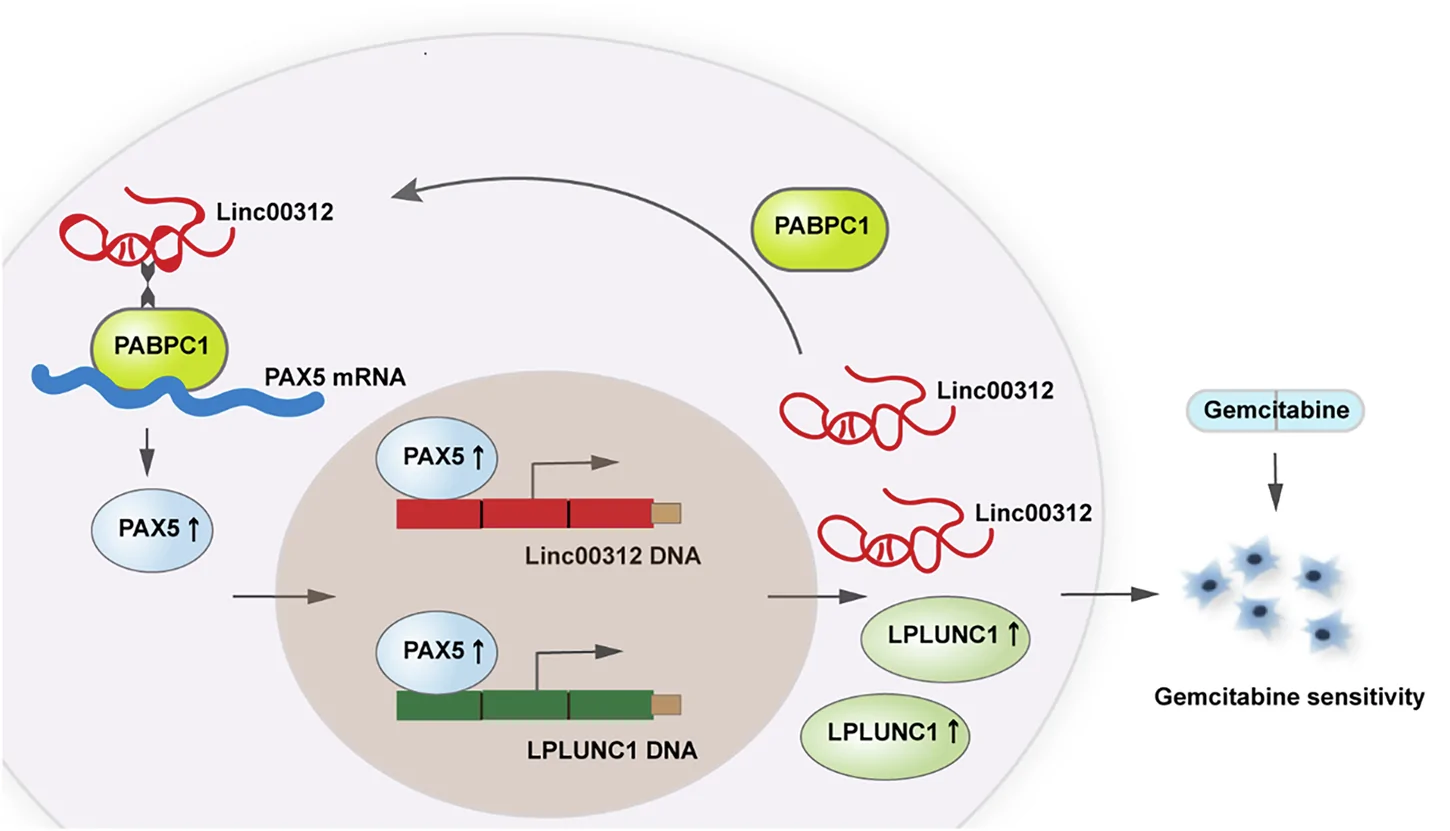
LINC00312 increases gemcitabine sensitivity in NPC via the PABPC1-PAX5-LPLUNC1 axis. LINC00312 recruits PABPC1 to increase the stability of PAX5 mRNA. PAX5 not only transcriptionally regulates LPLUNC1 to enhance NPC gemcitabine sensitivity, but also, in turn, transcriptionally activates LINC00312 to form a feedback axis that further increases gemcitabine sensitivity.

In conclusion, this study revealed that LINC00312 recruited PABPC1 in NPC to increase the stability of PAX5 mRNA. On the one hand, PAX5 transcriptionally regulates LPLUNC1 to regulate the sensitivity of NPC to gemcitabine. On the other hand, PAX5 in turn transcriptionally activated LINC00312, forming a feedback loop to further increase NPC gemcitabine sensitivity (Fig. 9). Our study depicted a novel molecular mechanism to overcome NPC resistance to gemcitabine.

## Methods

### Clinical samples

We collected 18 rhinitis samples and 30 NPC samples at Hunan Provincial Cancer Hospital (Changsha, China), with all patients having received no preoperative treatment. Additionally, 10 paired NPC tissues were collected before and after gemcitabine treatment. Furthermore, 20 gemcitabine-treated samples were categorized based on post-treatment disease progression into gemcitabine-resistant or gemcitabine-sensitive groups, with 10 samples in each group. Informed consent was acquired from each participant before inclusion in the study. All experiments were approved by the Independent Ethical Committee of the Institute of Clinical Pharmacology, Central South University (CTXY-140007-2) and were conducted in accordance with the Declaration of Helsinki.

### Cell culture

Normal nasopharyngeal epithelial cells NP69 and nasopharyngeal cancer cells CNE3, NPC/HK-1, and C666-1 were acquired from iCell Bioscience (Shanghai, China). NP69 cells were cultured in K-SFM medium (Invitrogen, Carlsbad, CA, USA), while all other cell lines were maintained in RPMI 1640 medium (Invitrogen, Carlsbad, CA, USA) supplemented with 10% fetal bovine serum (FBS; Gibco). All cells were incubated at 37 °C in a humidified atmosphere containing 5% CO_2_. Different concentrations of gemcitabine (0, 2, 4, 6, 8, 10 nM, MedChemExpress, Monmouth Junction, NJ, USA) were used to treat CNE3 and NPC/HK-1 cells to test the sensitivity of the above cells to gemcitabine.

### Cell transfection

LINC00312 (NR_024065.3), LPLUNC1 (NM_033197.3), and PAX5 (NM_016734.3) cDNA were cloned into the pcDNA3.1 plasmid to construct overexpression vectors. Its negative control (NC) refers to the empty control vector. shRNAs for knockdown of LINC00312, LPLUNC1, PAX5 and PABPC1 were acquired from GenePharma (Shanghai, China). Negative control for shRNA (shNC) represents a scrambled or non-targeting short hairpin RNA. Lipofectamine 3000 (Thermo Fisher Scientific, MA, USA) was used for transfection. The above plasmids and sequences were packaged into lentivirus by Genechem (Shanghai, China). CNE3 and NPC/HK-1 cells were infected with the above lentiviruses at a multiplicity of infection (MOI) of 30. The target sequences for all shRNAs were detailed as follows:

shLINC00312: 5′-GCTTTATCACTCTTGAATATT-3′;

shPAX5: 5′-CCCTCAGTATTCCTCGTACAA-3′;

shPABPC1: 5′-GCAAAGTATTTGTTGGACGAT-3′;

shLPLUNC1: 5′-GAAGGTCATCACAGCTAACAT-3′.

### Quantitative real-time PCR (RT-qPCR)

Total RNA from tissues and cells was extracted using RNAiso Plus reagent (Takara, Tokyo, Japan), and RNA was quantified using a micro-volume nucleic acid quantifier (Thermo Fisher Scientific). The PrimeScript RT kit (Takara, Tokyo, Japan) was implemented to reverse transcribe RNA into cDNA. RT-qPCR assay was performed using SYBR Premix ExTaq (Takara, Tokyo, Japan). Relative gene expression was tested using GAPDH as an internal reference and analyzed using the 2^-ΔΔCt^ method. The primers used in qPCR analysis were detailed as follows (5′-3′) :

LINC00312-Forward: 5’-CCCTGTGAGAGCAACAGGAG-3’;

LINC00312-Reverse: 5′-CTGTGCCACTTGGCTTCTCT-3′;

PAX5-Forward: 5′-ACTTGCTCATCAAGGTGTCAG-3′;

PAX5-Reverse: 5′-TCCTCCAATTACCCCAGGCTT-3′;

LPLUNC1-Forward: 5′-GGAGCTGCTAGTCAAGATCCC-3′;

LPLUNC1-Reverse: 5′-CTCAGTCGTCATGTGGAACTC-3′;

PABPC1-Forward: 5′-CCCTGTGAGAGCAACAGGAG-3′;

PABPC1-Reverse: 5′-CTGTGCCACTTGGCTTCTCT-3′;

GADPH-Forward: 5′-CCAGGTGGTCTCCTCTGA-3′;

GAPDH-Reverse: 5′-GCTGTAGCCAAATCGTTGT-3′.

### Western blot

RIPA high-performance lysate (Beyotime, Shanghai, China) was applied to extract proteins from cell and tissue samples. The extracted proteins were quantified using the BCA protein assay kit (Beyotime). SDS-PAGE gel was used to separate the proteins, and subsequently, the target proteins were transferred to PVDF membranes. The membranes were blocked, and then incubated with anti-LPLUNC1 (ab219098, Abcam), anti-PAX5 (ab109443, Abcam) and anti-PABPC1 (10970-1-AP, Proteintech), anti-β-actin (AC026, ABclonal) antibodies overnight. The next day, the membranes were incubated with the secondary antibodies at room temperature for 1 h. Then, ECL was added to the PVDF membranes for visualization.

### Cell counting kit-8 (CCK-8) assay

Cells transfected with the corresponding vectors were inoculated into 96-well plates (5 × 10^3^ cells/well). Then, 10 μL of CCK-8 solution (BioGLP, Montclair, CA, USA) was added to the 96-well plates to continue incubation for 2 h. Finally, a microplate reader was used to examine the OD value of each well at 450 nm.

### Colony formation assay

Cells transfected with the corresponding vectors were collected and resuspended in complete medium, and then inoculated in 6-well plates (0.8 × 10^3^ cells/well). After 14 days, the cells were fixed using 4% paraformaldehyde. The cells were washed and stained with crystal violet stain solution (Solarbio, China) for 20 min. Finally, the results of the experiment were photographed and recorded.

### Transwell assay

Cells transfected with the corresponding vectors were collected and inoculated into the upper chamber. Matrigel matrix (Corning, NY, USA) was pre-coated on the upper chamber membrane in the cell invasion assay. Subsequently, complete medium was added to the lower chamber. After the cells continued to be cultured for 48 h, the cells on the upper chamber membrane were fixed with 10% paraformaldehyde for 10 min and then stained with 5% crystal violet. Finally, electron microscopy (Leica, Wetzlar, Germany) was applied to observe cell invasion.

### Flow cytometry

The Annexin V-FITC/PI apoptosis detection kit (Beyotime) was utilized for apoptosis assessment. After the cells were collected, 500 μL of 1× binding buffer was applied to resuspend the cells, and the number of cells was not less than 5 × 10^5^. Subsequently, 1 μL of membrane-bound protein V-FITC and 5 μL of PI were added to the cells, followed by incubation for 5 min. Finally, flow cytometry (BD Biosciences, San Jose, CA, USA) was employed to detect apoptosis.

### Chromatin immunoprecipitation (ChIP)

The binding of PAX5 to LINC00312 or LPLUNC1 promoter was assessed by ChIP using the ChIP Kit (Bes5001, BersinBio, Guangzhou, China). Cells were fixed with 1% formaldehyde for 10 min for cross-linking. Cells were then lysed, and DNA was fragmented by ultrasound. Anti-PAX5 (ab109443, Abcam) or anti-IgG (ab172730, Abcam) antibody was added to the chromatin for immunoprecipitation at 4 °C overnight. The following day, protein A/G-agarose beads were added to the above mixture and incubated for 2 h. Chromatin was then eluted and purified. Finally, chromosome fragments were analyzed by PCR to determine the binding relationship between PAX5 and LINC00312 or LPLUNC1 promoter region. The primers used were detailed as follows:

LINC00312-BS1-Forward: 5′-CTATCTGGCATGGTGGCACAC-3′;

LINC00312-BS1-Reverse: 5′-GCACATGCATTCATGTATCC-3′;

LINC00312-BS2-Forward: 5′-TGAACATGGCCCATGGATGG-3′;

LINC00312-BS2-Reverse: 5′-GTATCTCATGATCTCCCTGTCC-3′;

LPLUNC1-BS1-Forward: 5′- GAATCCCTGGTCATGTGACC -3′;

LPLUNC1-BS1-Reverse: 5′- GAGCACTTGTGGACCAAAAG-3′.

### Dual-luciferase reporter assay

To explore the effect of PAX5 on LPLUNC1 and LINC00312 promoter activity, the LPLUNC1 or LINC00312 promoter, along with its mutant variants (LPLUNC1-WT, LPLUNC1-MUT, LINC00312-WT, LINC00312-BS1-MUT, LINC00312-BS2-MUT, and LINC00312-BS1&BS2-MUT), was then cloned into the psiCHECK2 vector (Promega, Madison, USA). Then, LPLUNC1-WT or LPLUNC1-MUT (LINC00312-WT or LINC00312-MUT) was co-transfected with shNC or shPAX5 into NPC/HK-1 or CNE3 cells by Lipofectamine 3000 (Invitrogen). After 48 h of incubation, luciferase activity was measured by the Dual-Luciferase Reporter Assay Kit (E1910, Promega).

### RNA pull-down

RNA pull-down assays were performed using the RNA-Protein Pull Down Kit (Sangon, Shanghai, China). Briefly, 1–2 μg of biotin-labeled LINC00312 or PAX5 probe was incubated with whole-cell lysate for 30 min. Subsequently, pre-post-treated streptavidin magnetic beads were added to the above mixture and incubated for 2 h. Then, a magnetic rack was adopted to collect the magnetic beads and remove the supernatant. The magnetic beads were washed with protein elution buffer for 2 h. Finally, the binding of LINC00312 or PAX5 mRNA to PABPC1 protein was detected by western blot.

LINC00312 truncations (1-2287 nt, 1-1000 nt, 1-2000 nt, 2001-2887 nt) or PAX5 mRNA truncations (3′UTR, CDS, 5′UTR) were obtained from Goldrex (Nanjing, China). The above truncations and mutants were transfected into 293 T cells. Subsequently, the RNA pull-down procedure was performed as described above.

### RNA immunoprecipitation (RIP)

NPC cells were lysed with RIP lysis buffer. Protein A/G magnetic beads conjugated with anti-IgG (ab172730, Abcam), anti-HNRNPA1 (11176-1-AP, Proteintech), anti-NONO (11058-1-AP, Proteintech), anti-PABPC1 (10970-1-AP, Proteintech), anti-FUS (PA5-52610, Thermo Fisher Scientific), anti-PUM2 (ab92390, Abcam), anti-EIF4B (PA5-92449, Thermo Fisher Scientific), anti-MBNL1 (ab309348, Abcam), anti-KHSRP (ab264265, Abcam), anti-RBMX (#14794, Cell Signaling Technology), or anti-SFRS1 (12929-2-AP, Proteintech) were incubated with cell lysates overnight at 4 °C. Besides, HA tagged RBMY1A1, Flag tagged SFRS13A or Myc tagged KHDRBS3 was transfected into CNE3 or NPC/HK-1 cells, and the above lysed supernatants were incubated with anti-HA-antibody (ab9110, Abcam), anti-Flag-antibody (ab205606, Abcam), anti-Myc-antibody (ab32, Abcam), respectively. Then, proteinase K buffer was added to the RNA-protein complexes. Finally, RNA was extracted using the phenol-chloroform method. RT-qPCR was employed to determine the interaction between LINC00312 or PAX5 mRNA and PABPC1 protein.

### Fluorescence in situ hybridization (FISH) and immunofluorescence (IF)

FISH and IF were used to analyze the co-localization of LINC00312 and PABPC1 in cells. The LINC00312 FISH probe was designed and synthesized by Sangon, and the probe was denatured in a 75 °C water bath. Cells were seeded on slides and fixed and permeabilized. Subsequently, cells were incubated with the LINC00312 probe in hybridization buffer overnight. Anti-PABPC1 antibody (10970-1-AP, Proteintech) was added to continue the incubation. Then, the cells were labeled with Alexa Fluor Plus 488 goat anti-rabbit IgG secondary antibody for 1 h at room temperature. DAPI was adopted to stain the nuclei. Finally, photographs were taken using a laser scanning confocal microscope (LSM 900, Zeiss, Jena, Germany). Pearson’s correlation coefficient was calculated as the primary metric to evaluate the degree of overlap between the two fluorescence channels. The analysis was performed on at least 10 independent cell images per experiment, with three biological replicates. All images were processed with uniform parameters and without nonlinear adjustments to preserve the original signal distribution.

### RNA stability assay

NPC cells were inoculated in 6-well plates, and each group of cells was transfected with the corresponding vector. 5 μg/mL actinomycin D (Selleck Chemicals, Houston, TX, USA) was employed to treat the cells for different times (0, 4, 8 h). Finally, cells were collected, and RNA was extracted for analysis using RT-qPCR.

### Animal experiments

BALB/c nude mice aged 4–5 weeks were purchased from SJA (Changsha, China) and cared for in an SPF-rated environment. Mice were randomly divided into 5 groups, with 6 mice in each group. Subsequently, 1 × 10^6^ NPC cells transfected with control lentivirus, LINC00312-ovexepressing lentivirus, sh-NC lentivirus, shPAX5 lentivirus, LINC00312 plus shPAX5 lentivirus, were injected subcutaneously into mice. After 5 days of transplantation, mice were regularly injected intraperitoneally with gemcitabine (50 mg/kg) every other day. Tumor volume was tested every 5 days and calculated by (length×width^2^)/2. All mice were anesthetized with carbon dioxide and euthanized by cervical dislocation at 25 days after transplantation. Finally, the tumor tissues were divided into two parts; one part was placed in formalin for pathological examination, and the other part was placed in liquid nitrogen.

### Immunohistochemical (IHC)

Tumor tissues were embedded and sectioned. Subsequently, sections were deparaffinized, and sodium citrate solution (Biosharp, Hefei, China) was added to the sections for antigen retrieval. After the sections were blocked with blocking solution, anti-PAX5 (ab109443, Abcam), anti-LPLUNC1 (ab219098, Abcam), and anti-Ki-67 (27309-1-AP, Proteintech) primary antibodies were added to the sections and incubated at 4 °C overnight. Then, the HRP-labeled secondary antibody was added to the section and incubated for 30 min. Sections were then stained with the DAB kit (Biosharp) and captured by a microscope.

### Bioinformatics analysis

RNA sequencing data from Gene Expression Omnibus (GEO) database (https://www.ncbi.nlm.nih.gov/geo/) under accession numbers GSE12452 and GSE53819 were used to analyze the differential expression of LPLUNC1 and PAX5 in NPC patients, as well as the correlation between LPLUNC1 and PAX5/LINC00312 levels in NPC patients. The Rbpdb database (http://rbpdb.ccbr.utoronto.ca/index.php) is utilized to predict RBPs that interact with LINC00312 or PAX5 mRNA.

### Statistical analysis

Data were analyzed using GraphPad Prism 10.0 software. All experiments were replicated at least three times, and data were expressed as mean ± standard deviation. Differences between any two groups were assessed using the Student’s t-test, and significant differences between multiple groups were estimated using one-way analysis of variance (ANOVA) with Tukey’s post hoc test. *P* < 0.05 meant the difference was significant.

## Supplementary information

Supplementary information

Supplementary Materials - uncropped wb.

## Data Availability

The data used to support the findings of this study are included within the article and supplementary file.
