# Supplementary material for: LINC00312 increases gemcitabine sensitivity in nasopharyngeal carcinoma by recruiting PABPC1 and modulating PAX5-mediated transcription of LPLUNC1
Source: NPJ Precis Oncol. 2026 Jun 17;10:365. doi: 10.1038/s41698-026-01542-7 (PMC13631224; doi:10.1038/s41698-026-01542-7)
Supplement: Supplementary file 1 [file 41698_2026_1542_MOESM1_ESM.pdf]

Supplementary Materials

Supplementary Figure and Figure legends

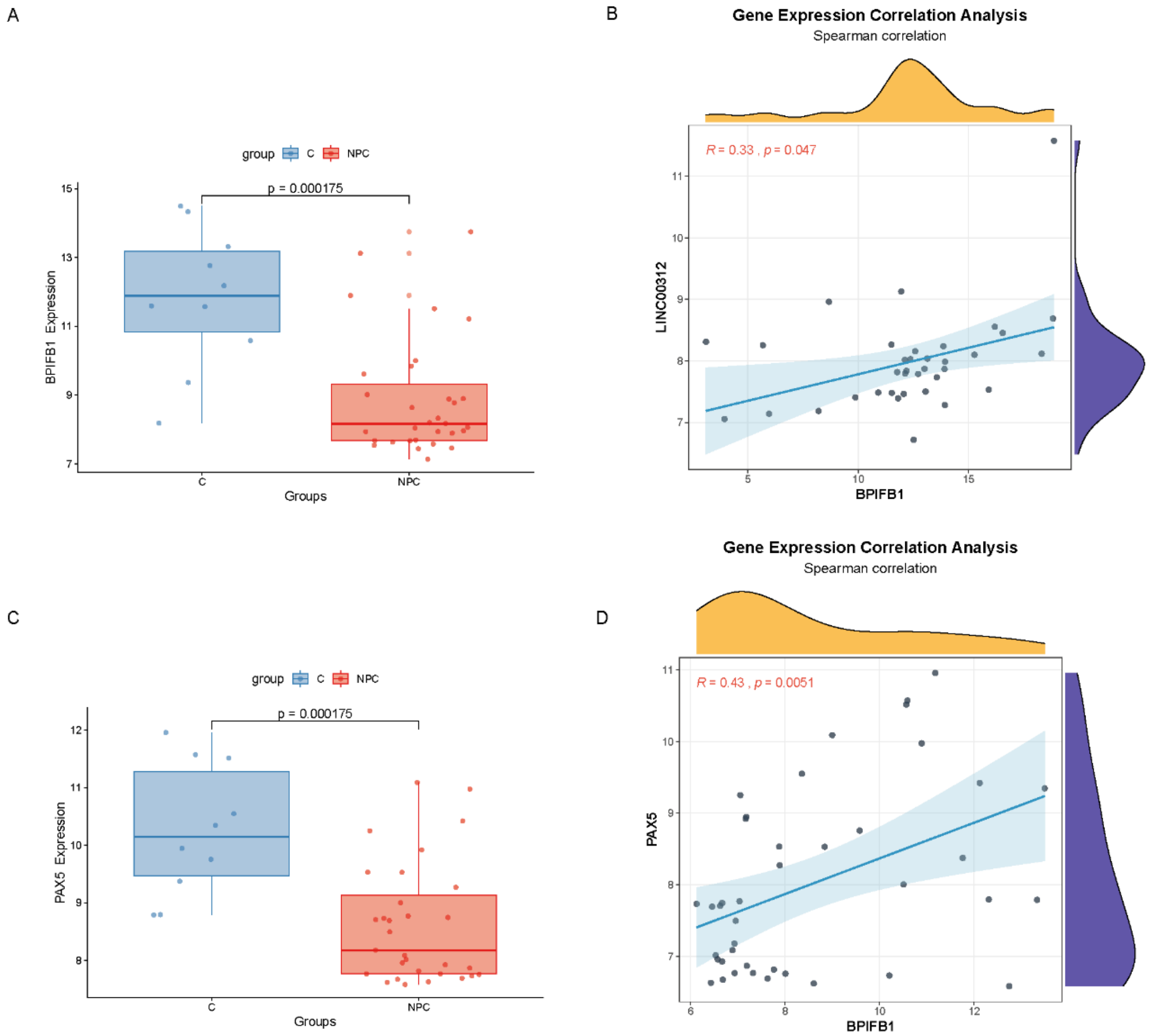

Supplementary Figure 1. GEO database analysis validates the co-downregulation and positive correlation among LINC00312, PAX5, and LPLUNC1 in NPC

(A) Analysis of LPLUNC1 expression in NPC versus normal tissues from the GEO database from GSE12452. (B) Correlation analysis based on GEO database data from GSE53819 shows positive associations between the expression levels of LINC00312 and LPLUNC1 in NPC clinical samples. (C) Analysis of PAX5 expression in NPC versus normal tissues from the GEO database from GSE12452. (D) Correlation analysis based on GEO database data from GSE12452 shows positive associations between the expression levels of PAX5 and LPLUNC1 in NPC clinical samples. **p*<0.05, ***p*<0.01, ****p*<0.001.

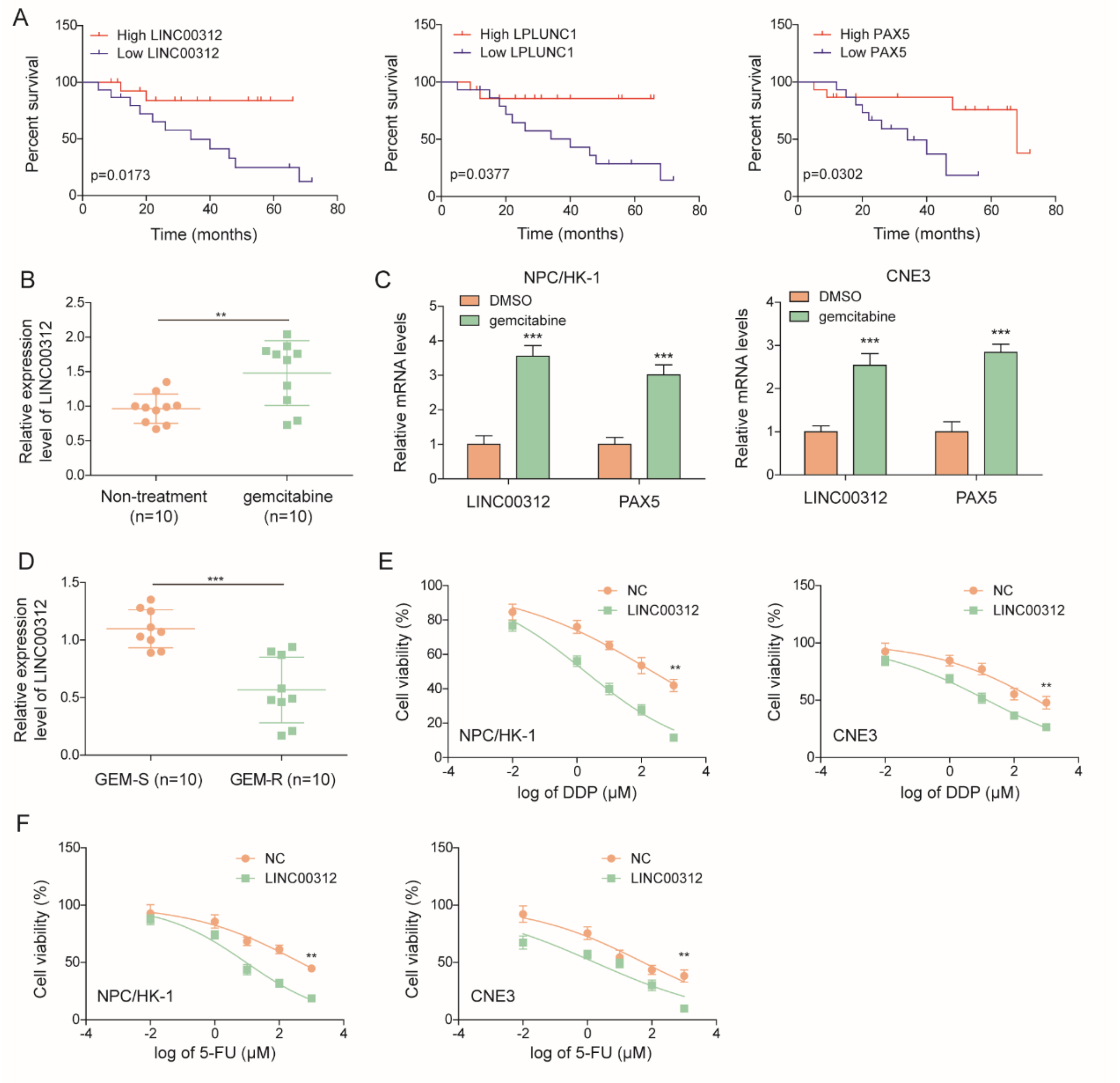

Supplementary Figure 2. Gemcitabine treatment upregulates the expression of LINC00312 in NPC tissues and cells, and impact of LINC00312 overexpression on chemoresistance to cisplatin and 5-FU in NPC cells. (A)Kaplan-Meier survival analysis of 5-year overall survival for the 30 NPC patients. Patients were stratified into high- and low-expression groups based on the median expression levels of LINC00312, PAX5, and LPLUNC1. (B) RT-qPCR analyses showed that the expression levels of LINC00312 in NPC samples before and after gemcitabine treatment ($n = 10$). NPC/HK-1 and CNE3 cells were treated with gemcitabine. (C) RT-qPCR determined the mRNA levels of LINC00312 and PAX5 ($n = 3$). (D) RT-qPCR analyses revealed that the expression levels of LINC00312 in gemcitabine-resistant and sensitive tissues ($n = 10$ per group). (E-F) NPC/HK-1 and CNE3 cells were transfected with NC or LINC000312,

then treated with DDP or 5-FU. CCK-8 determined the cell viability in each group (n = 3). *p<0.05, **p<0.01, ***p<0.001.

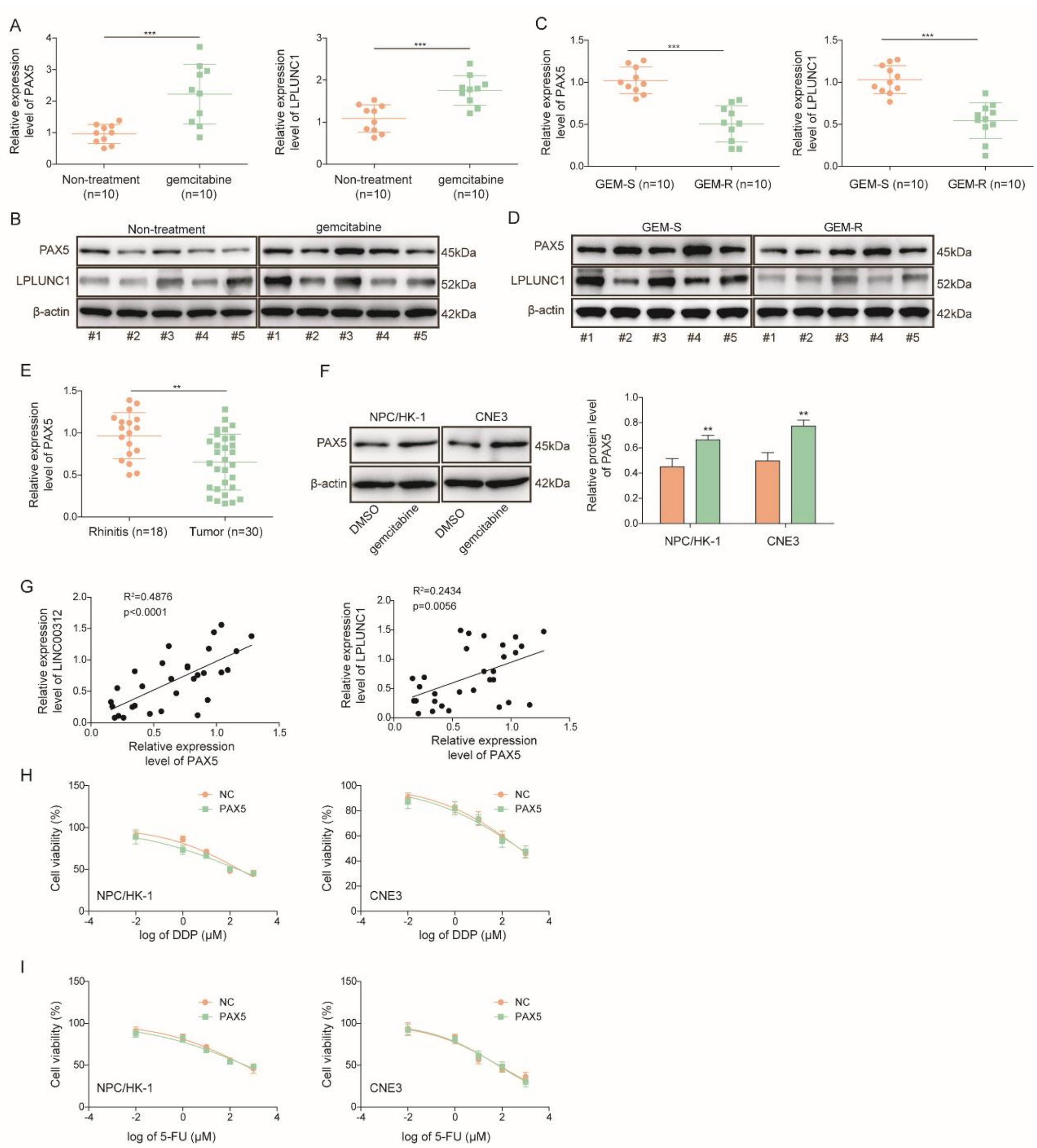

Supplementary Figure 3. PAX5 and LPLUNC1 expression is associated with gemcitabine treatment and response in NPC patients, and impact of PAX5 overexpression on chemoresistance to cisplatin and 5-FU in NPC cells.

(A) RT-qPCR and (B) Western blot (WB) analyses showed that the expression levels of PAX5 and LPLUNC1 in post-treatment and untreated NPC tissues. (C) RT-qPCR and (D) WB analyses revealed that the expression levels of PAX5 and LPLUNC1 in

gemcitabine-resistant and sensitive tissues. (E) RT-qPCR was applied to assess the expression of PAX5 in NPC tissues (*n* = 30) and rhinitis tissues (*n* = 18). NPC/HK-1 and CNE3 cells were treated with gemcitabine. (F) Western blot confirmed the protein level of PAX5 (*n* = 3). (G) Pearson correlation analysis performed on the 30 NPC tissue samples revealed significant positive correlations between the expression levels of PAX5 and LINC00312, and between PAX5 and LPLUNC1. (H-I) CCK-8 determined the cell viability of NPC/HK-1 and CNE3 cells transfected with NC or PAX5 and treated with DDP or 5-FU (*n* = 3). *$p<0.05$, **$p<0.01$, ***$p<0.001$.
