# Supplementary material for: LINC00312 increases gemcitabine sensitivity in nasopharyngeal carcinoma by recruiting PABPC1 and modulating PAX5-mediated transcription of LPLUNC1
Source: NPJ Precis Oncol. 2026 Jun 17;10:365. doi: 10.1038/s41698-026-01542-7 (PMC13631224; doi:10.1038/s41698-026-01542-7)

Supplementary Materials

The unedited Western blot bands.

Figure 1D

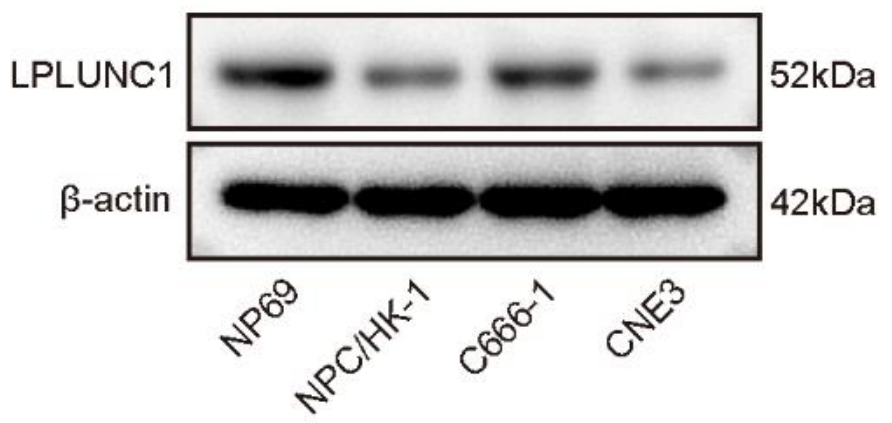

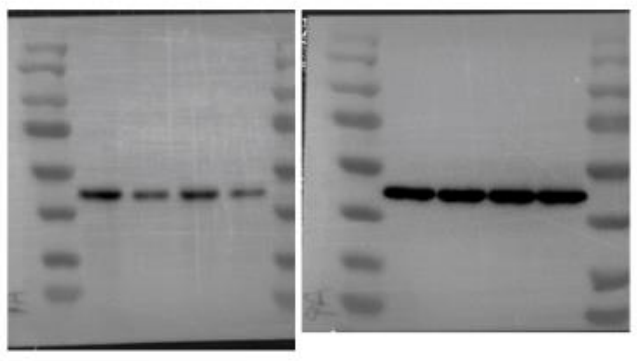

Figure 3B

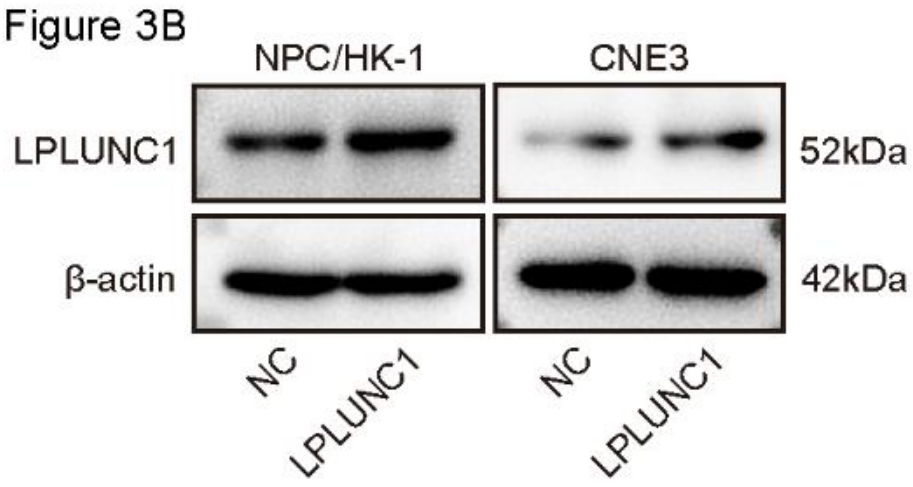

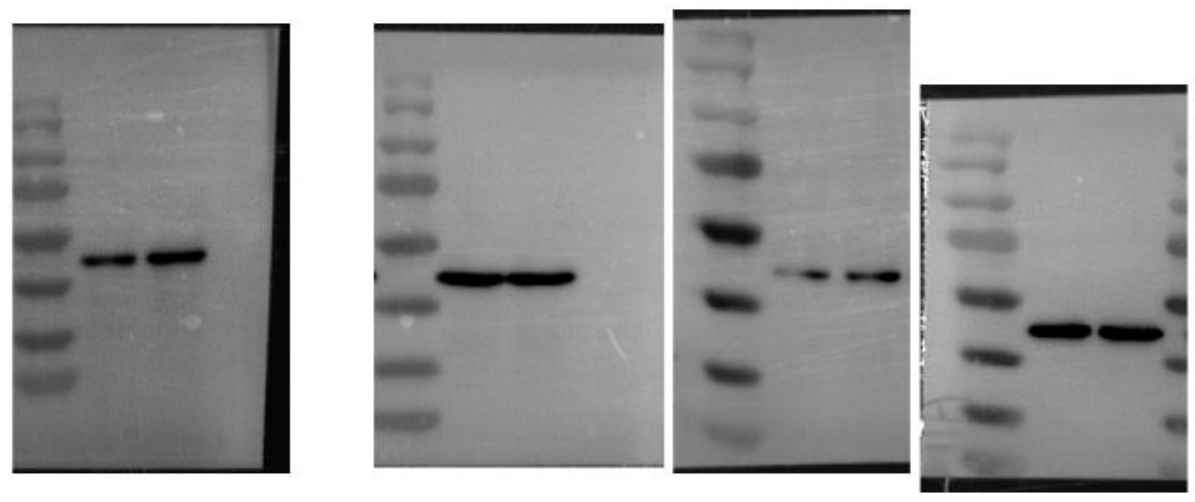

Figure 4

B

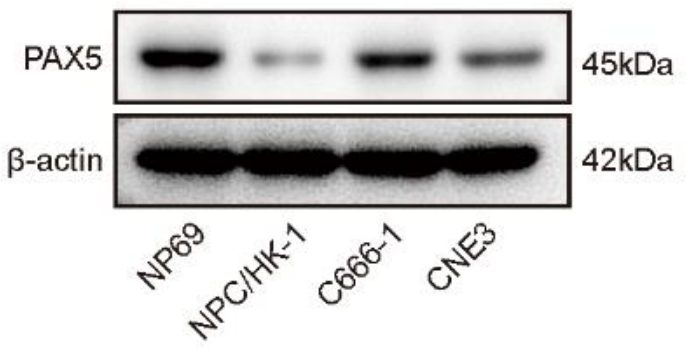

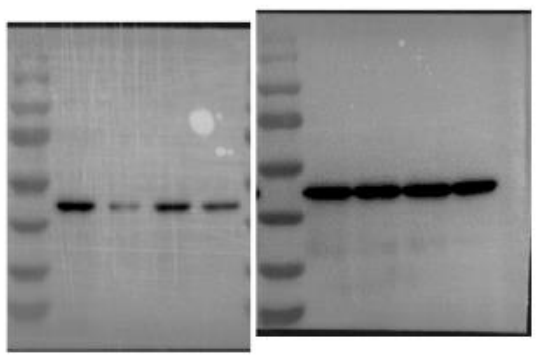

D

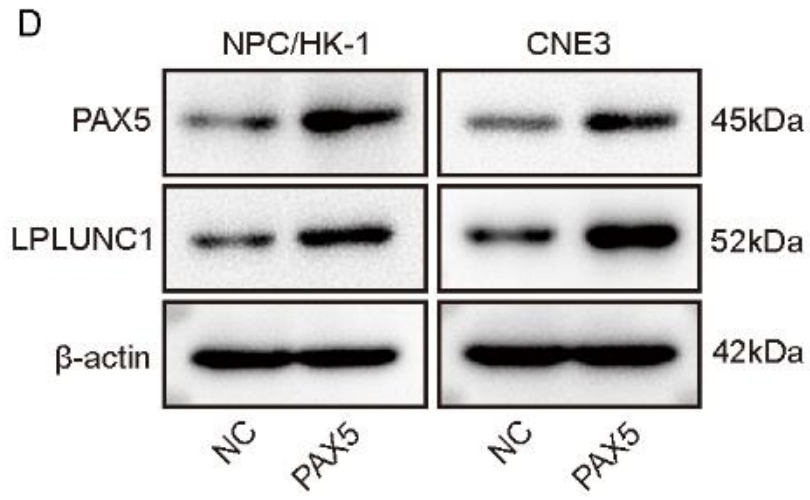

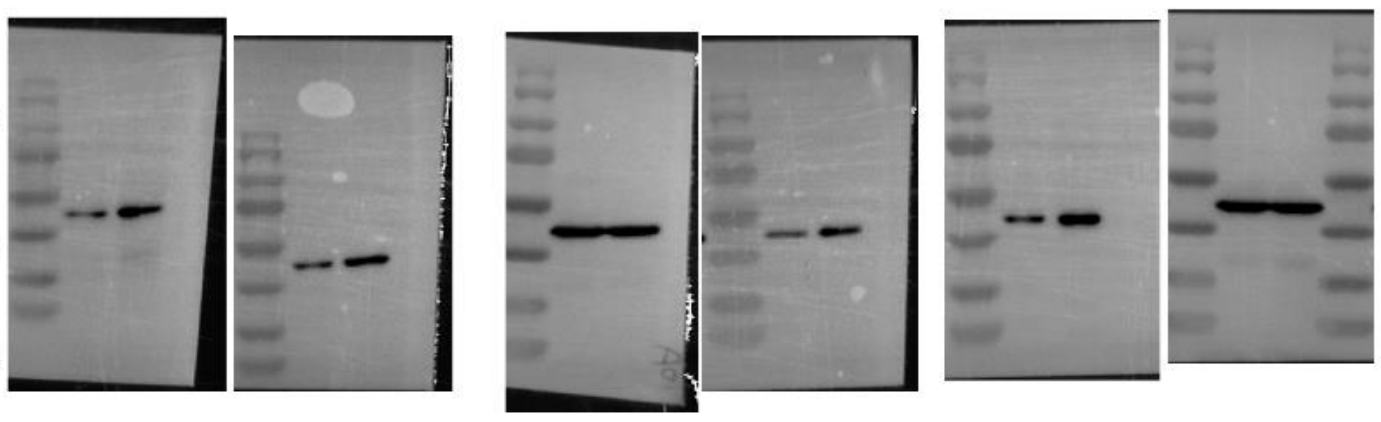

Figure 5B

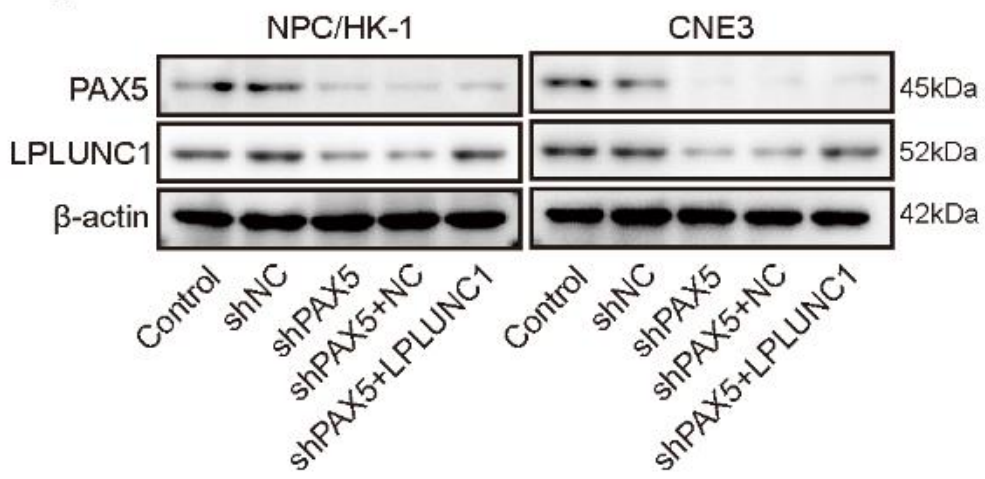

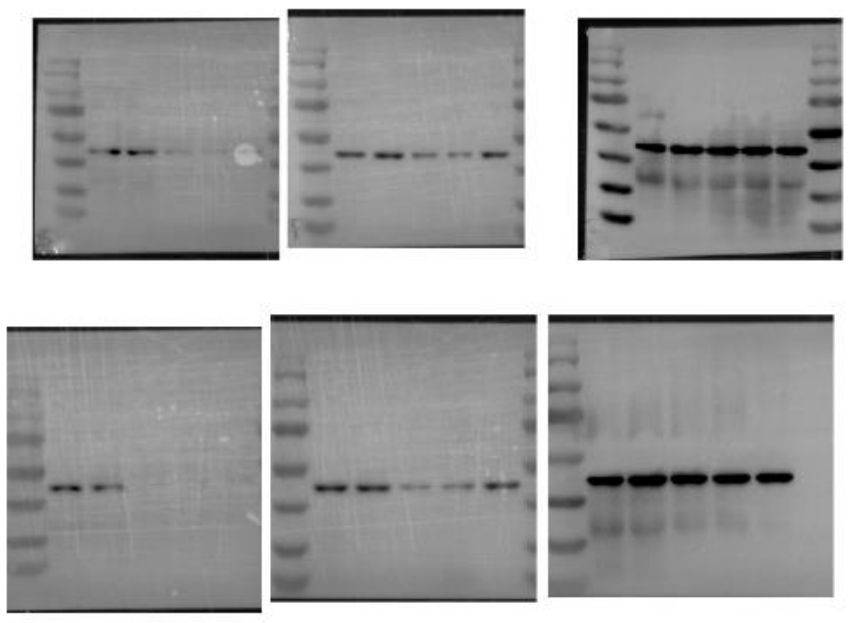

Figure 6

C

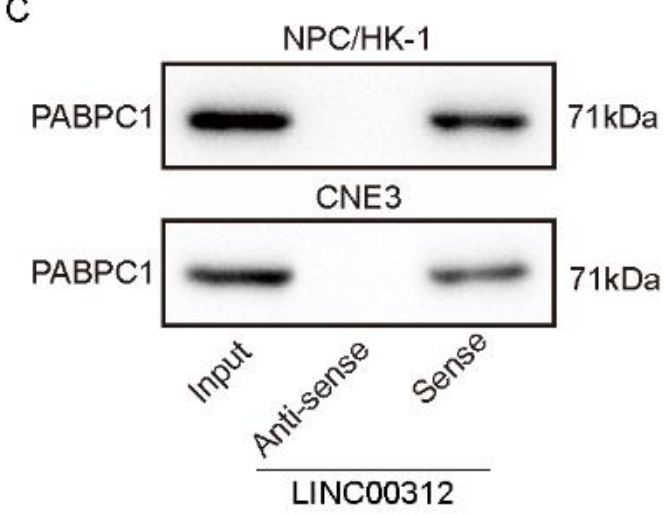

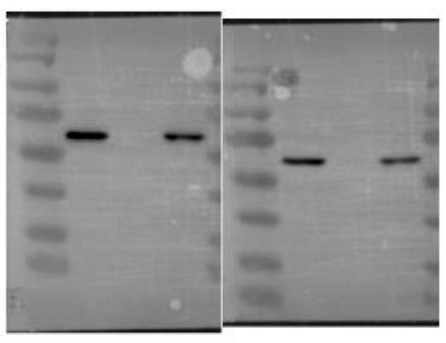

Figure 6

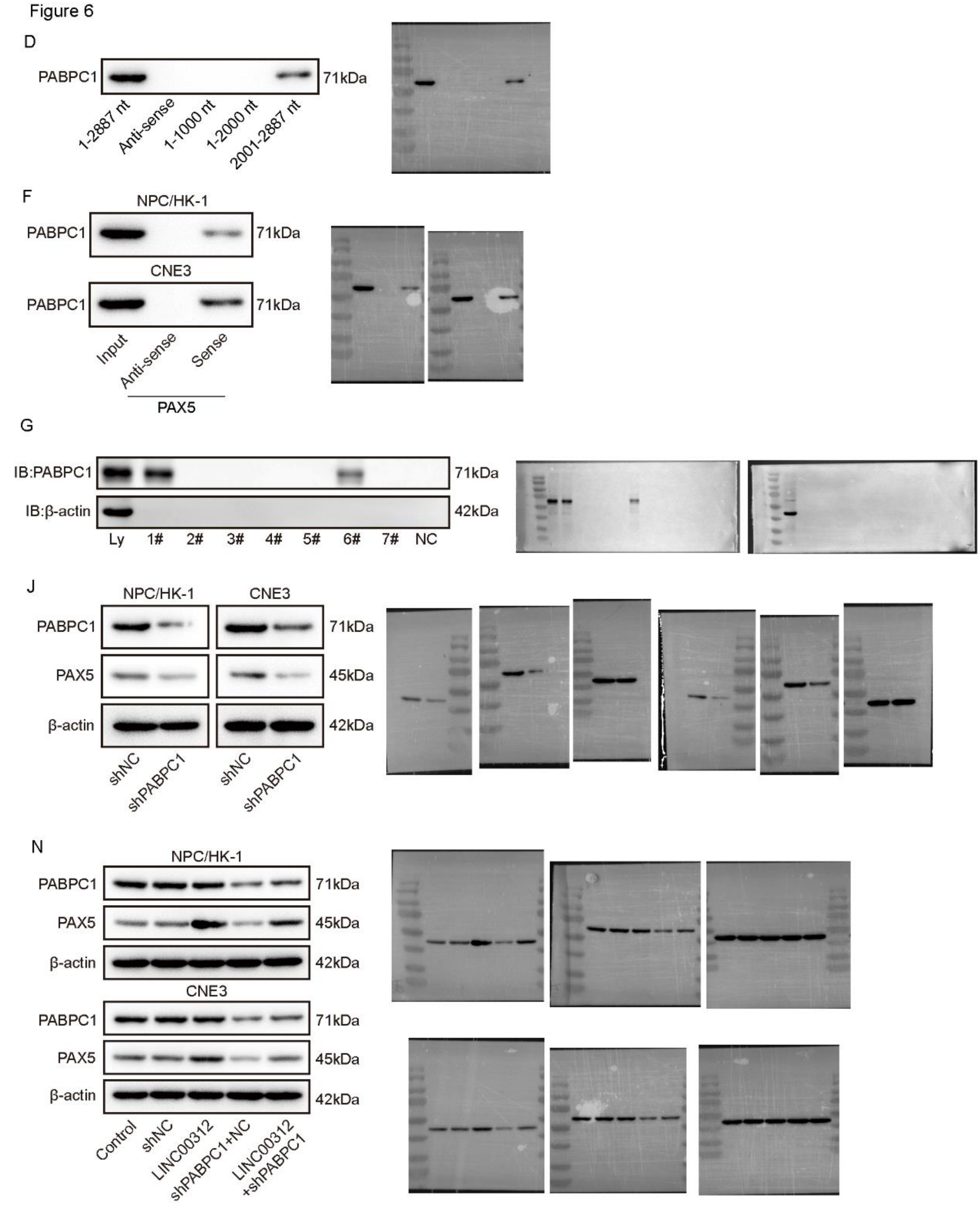
D
PABPC1
71kDa
1-2887 nt
Anti-sense
1-1000 nt
1-2000 nt
2001-2887 nt
F
NPC/HK-1
PABPC1
71kDa
CNE3
PABPC1
71kDa
Input
Anti-sense
Sense
PAX5
G
IB:PABPC1
71kDa
IB:β-actin
42kDa
Ly
1#
2#
3#
4#
5#
6#
7#
NC
J
NPC/HK-1
CNE3
PABPC1
71kDa
PAX5
45kDa
β-actin
42kDa
shNC
shPABPC1
shNC
shPABPC1
N
NPC/HK-1
PABPC1
71kDa
PAX5
45kDa
β-actin
42kDa
CNE3
PABPC1
71kDa
PAX5
45kDa
β-actin
42kDa
Control
shNC
LINC00312
shPABPC1+NC
LINC00312
+shPABPC1

Figure 7F

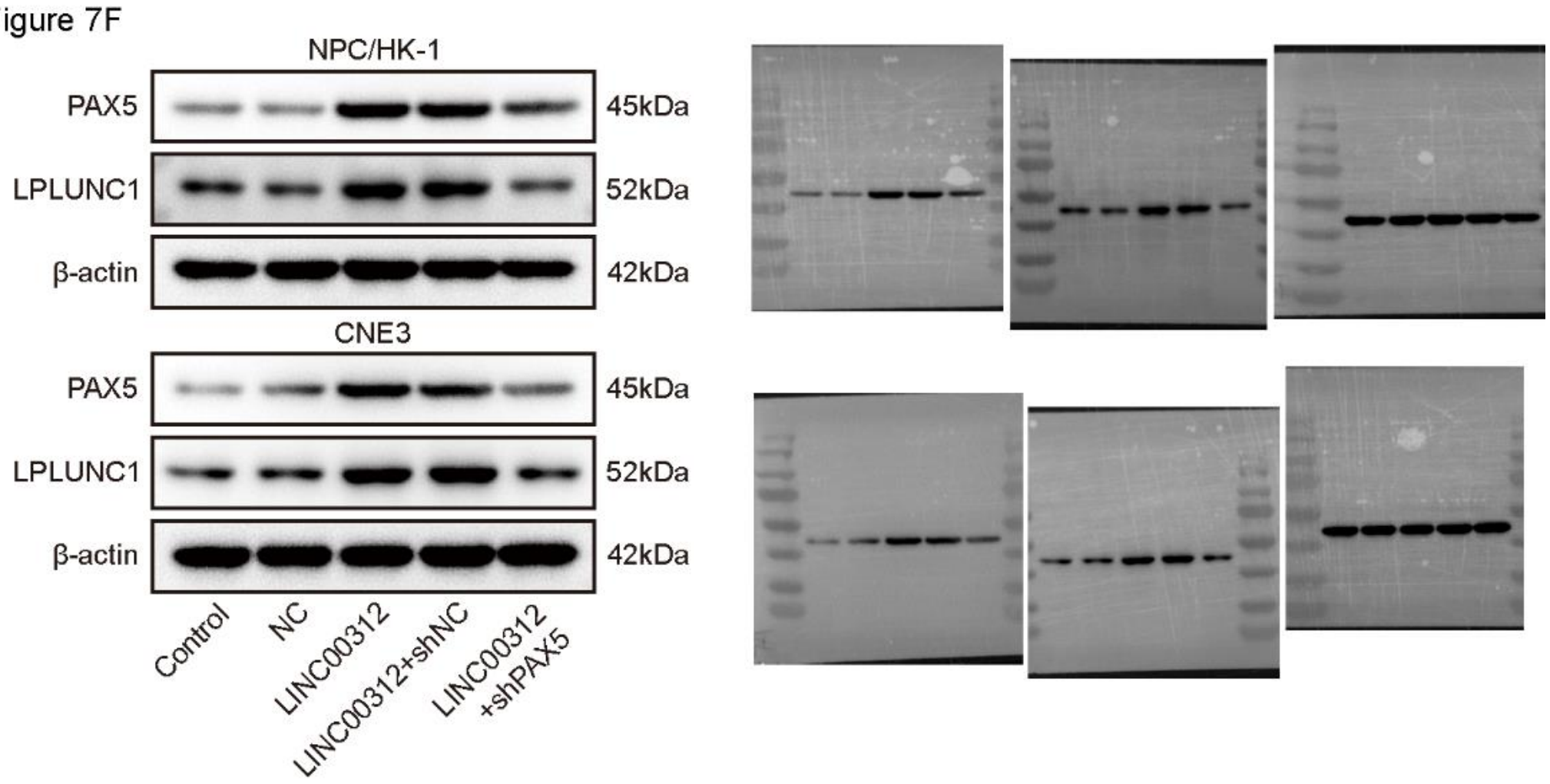

Figure 8F

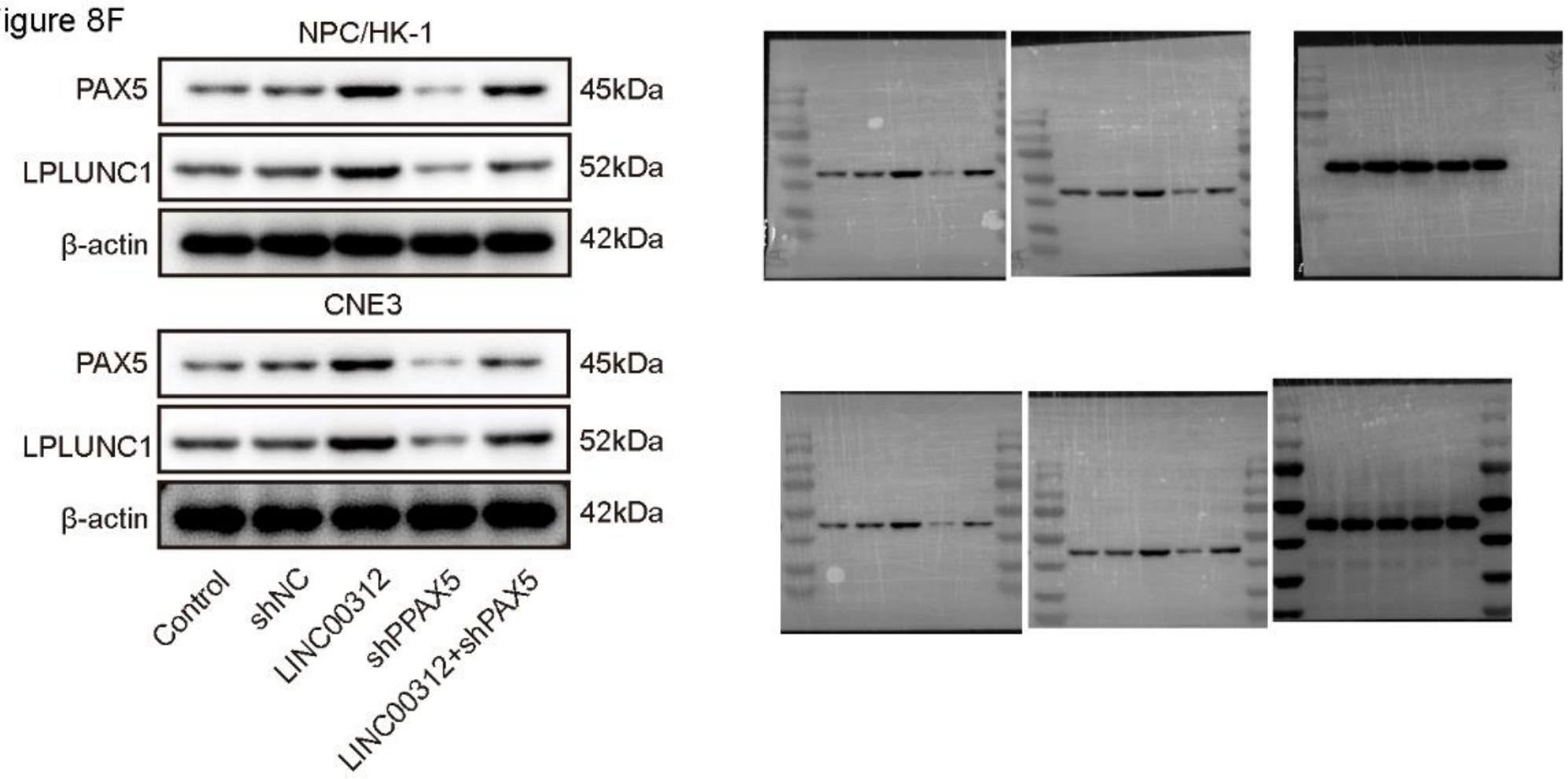

Figure S3

B

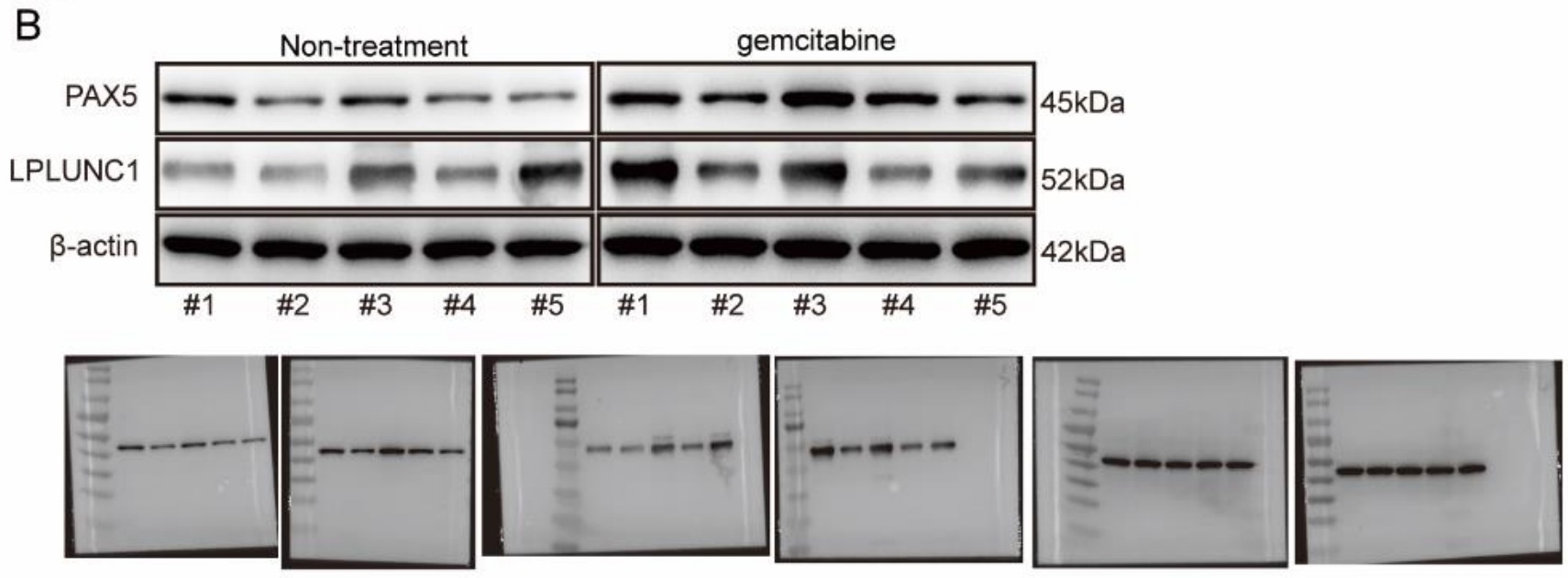

D

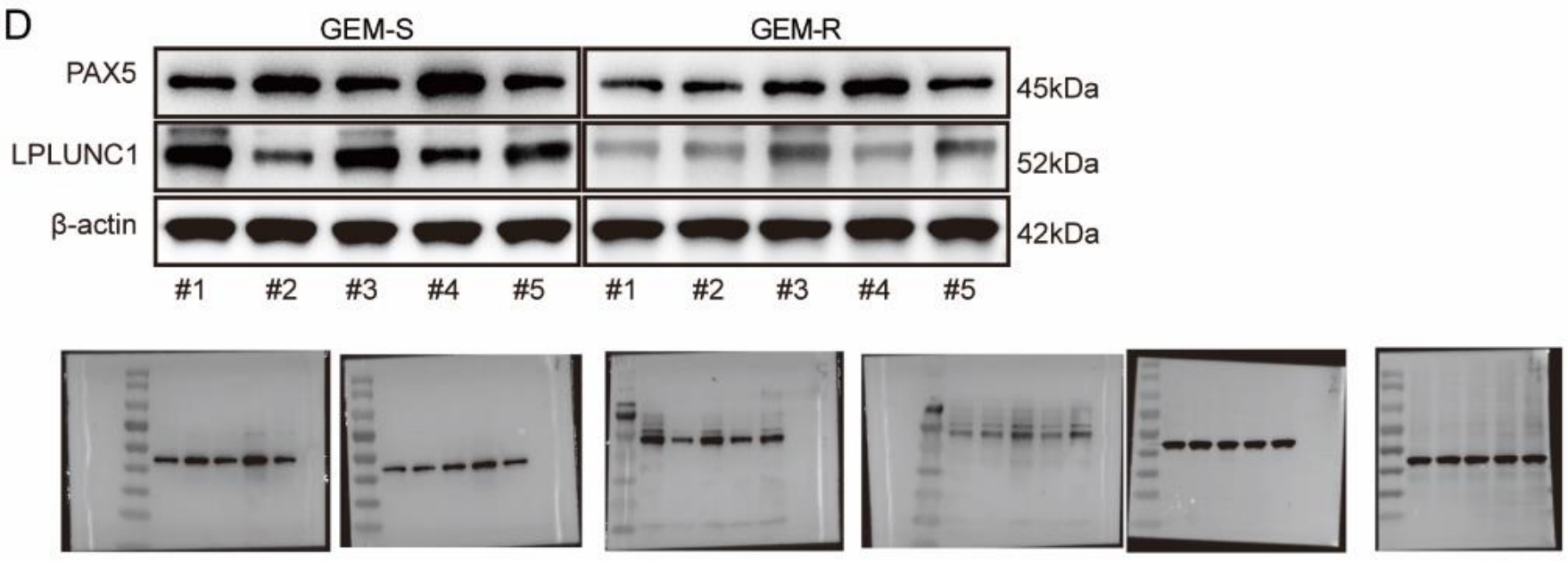

F

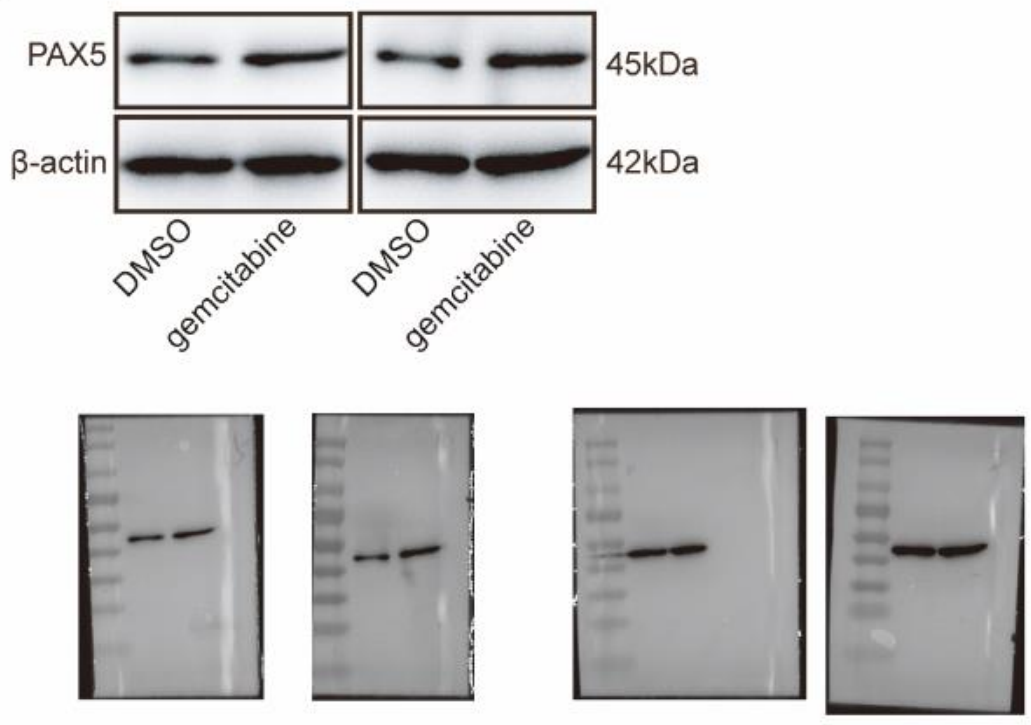

Supplement: Supplementary file 2 [file 41698_2026_1542_MOESM2_ESM.pdf]
